# Structural basis of the correct subunit assembly, aggregation, and intracellular degradation of nylon hydrolase

**DOI:** 10.1038/s41598-018-27860-w

**Published:** 2018-06-27

**Authors:** Seiji Negoro, Naoki Shibata, Young-Ho Lee, Ikki Takehara, Ryo Kinugasa, Keisuke Nagai, Yusuke Tanaka, Dai-ichiro Kato, Masahiro Takeo, Yuji Goto, Yoshiki Higuchi

**Affiliations:** 1 Department of Applied Chemistry, Graduate School of Engineering, University of Hyogo, Himeji, 671-2280 Japan; 2 Department of Picobiology, Graduate School of Life Science, University of Hyogo, Kamigori-cho, Ako-gun, 678-1297 Japan; 3 RIKEN Harima Institute, SPring-8 Center, Sayo-cho, Sayo-gun, 679-5148 Japan; 4 Institute for Protein Research, Osaka University, Suita, 565-0871 Japan; 5 Graduate School of Science and Engineering, Kagoshima University, Kagoshima, Japan

## Abstract

Nylon hydrolase (NylC) is initially expressed as an inactive precursor (36 kDa). The precursor is cleaved autocatalytically at Asn266/Thr267 to generate an active enzyme composed of an α subunit (27 kDa) and a β subunit (9 kDa). Four αβ heterodimers (molecules A-D) form a doughnut-shaped quaternary structure. In this study, the thermostability of the parental NylC was altered by amino acid substitutions located at the A/D interface (D122G/H130Y/D36A/L137A) or the A/B interface (E263Q) and spanned a range of 47 °C. Considering structural, biophysical, and biochemical analyses, we discuss the structural basis of the stability of nylon hydrolase. From the analytical centrifugation data obtained regarding the various mutant enzymes, we conclude that the assembly of the monomeric units is dynamically altered by the mutations. Finally, we propose a model that can predict whether the fate of the nascent polypeptide will be correct subunit assembly, inappropriate protein-protein interactions causing aggregation, or intracellular degradation of the polypeptide.

## Introduction

Protein-protein interactions leading to correct subunit assembly play important roles in protein stability or various biological functions, which could include the following: i) regulation of enzyme activities by the allosteric effect, ii) generation of an active center by utilizing several catalytic/substrate-binding residues located in different subunits, and iii) generation of a multifunctional enzyme by integrating several functions initially compartmentalized in different enzyme subunits into a single oligomeric enzyme. In addition, the stability of a monomeric protein is generally improved by the oligomerization of the protein^1–3^. An appropriate protein-protein interaction results in correct subunit assembly, whereas an inappropriate interaction causes the proteins to aggregate. The formation of insoluble protein aggregates and the aggregates themselves, as in the case of the amyloidogenesis of amyloid β peptides, can lead to various diseases, including diseases of the nervous system^4,5^. Therefore, it is important to analyze the molecular basis of protein assembly to discriminate between the correct oligomer formation required for a functional enzyme and incorrect associations leading to the aggregation of the monomeric units.

**Figure 1:**
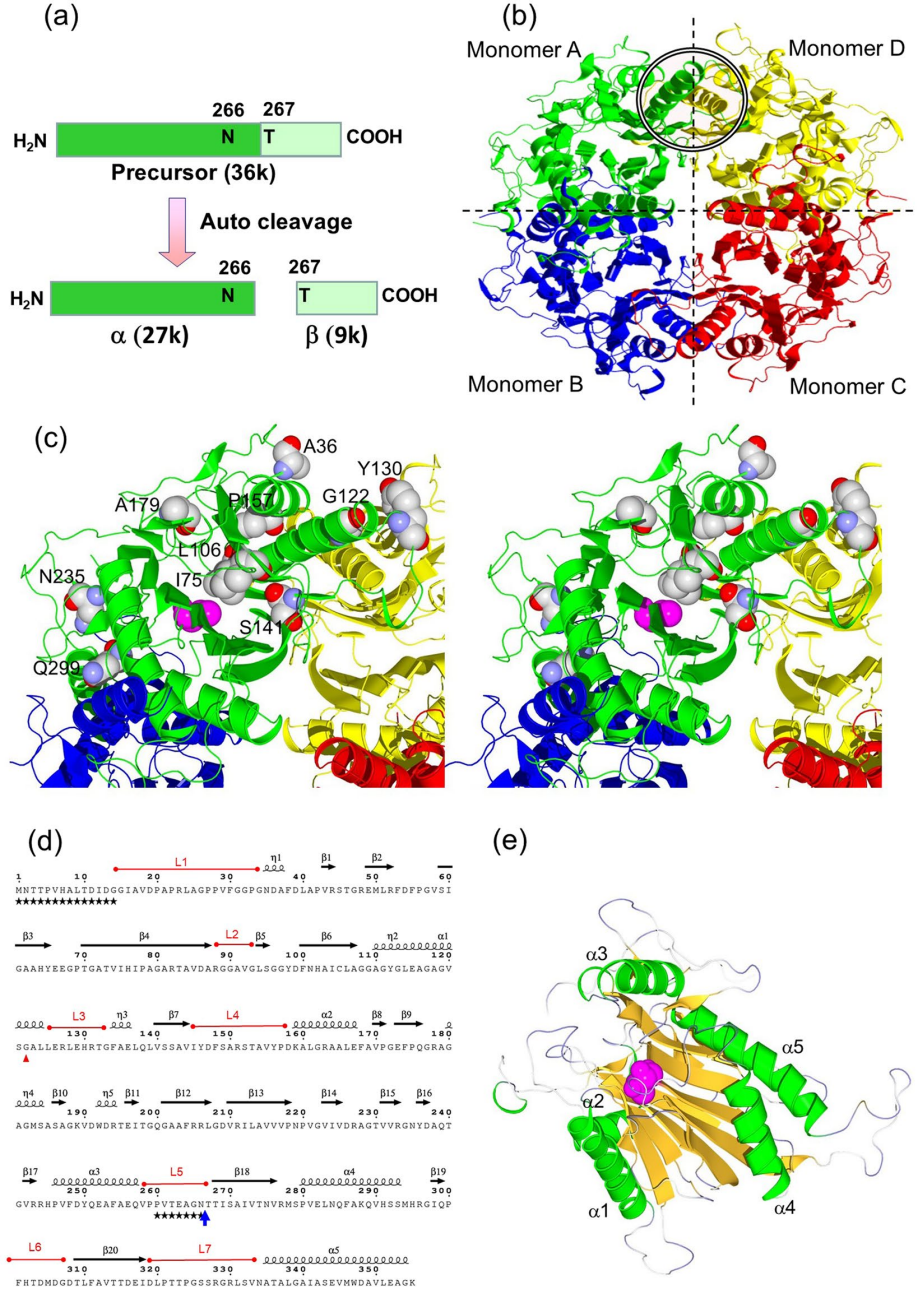
Structure of NylC. (**a**) Autocleavage of the NylC precursor between Asn266 and Thr267 generates an active enzyme composed of α and β subunits. (**b**) The quaternary structure of NylC_p2_ is shown as a *ribbon model*, with different colors highlighting the individual monomer molecules A (*green*), B (*blue*), C (*red*), and D (*yellow*). The positions of helix α1 in monomers A and D are marked by circles with double solid lines. (**c**) An enlarged view of monomer A and its interfaces with the adjacent monomers B and D in the NylC_p2_-G^122^Y^130^A^36^Q^263^ mutant enzyme is shown as a stereo diagram. Three mutated residues (Ala36, Gly122, and Tyr130) integrated into wild-type NylC_p2_ and seven additionally mutated residues (Ile75, Leu106, Ser141, Pro157, Ala179, Asn235, and Gln299) obtained by random mutagenesis are shown as *space-filling models*. The catalytic residue Thr267 (in the N-terminus of the β subunit) is shown in *reddish purple*. (**d**) The secondary structure of NylC_p2_-G^122^ mutant was illustrated by the program “ENDscript” (http://espript.ibcp.fr/ESPript/ENDscript/index.php). The amino acid sequence is shown using *one-letter* codes. The autocleaved site (N266/T267) and the D122G mutation are shown as a *blue vertical arrow* and a *red triangle*, respectively. Five α-helices (α1–α5) and five 3_10_-helices (η1–η5) are shown as *black coils*. Twenty β-strands (β1–β20) are shown as horizontal *black arrows*. The seven loop regions (L1-L7) described in the text are shown in *red*. Due to poor electron density, the conformations of the amino acid residues marked by stars could not be determined. (**e**) Subunit structure of the NylC_p2_-G^122^ mutant shown as a *ribbon model*. The α-helices and β-strands are colored in *green* and *orange*, respectively. Catalytic residue Thr267 is shown in *reddish purple*.

**Figure 2:**
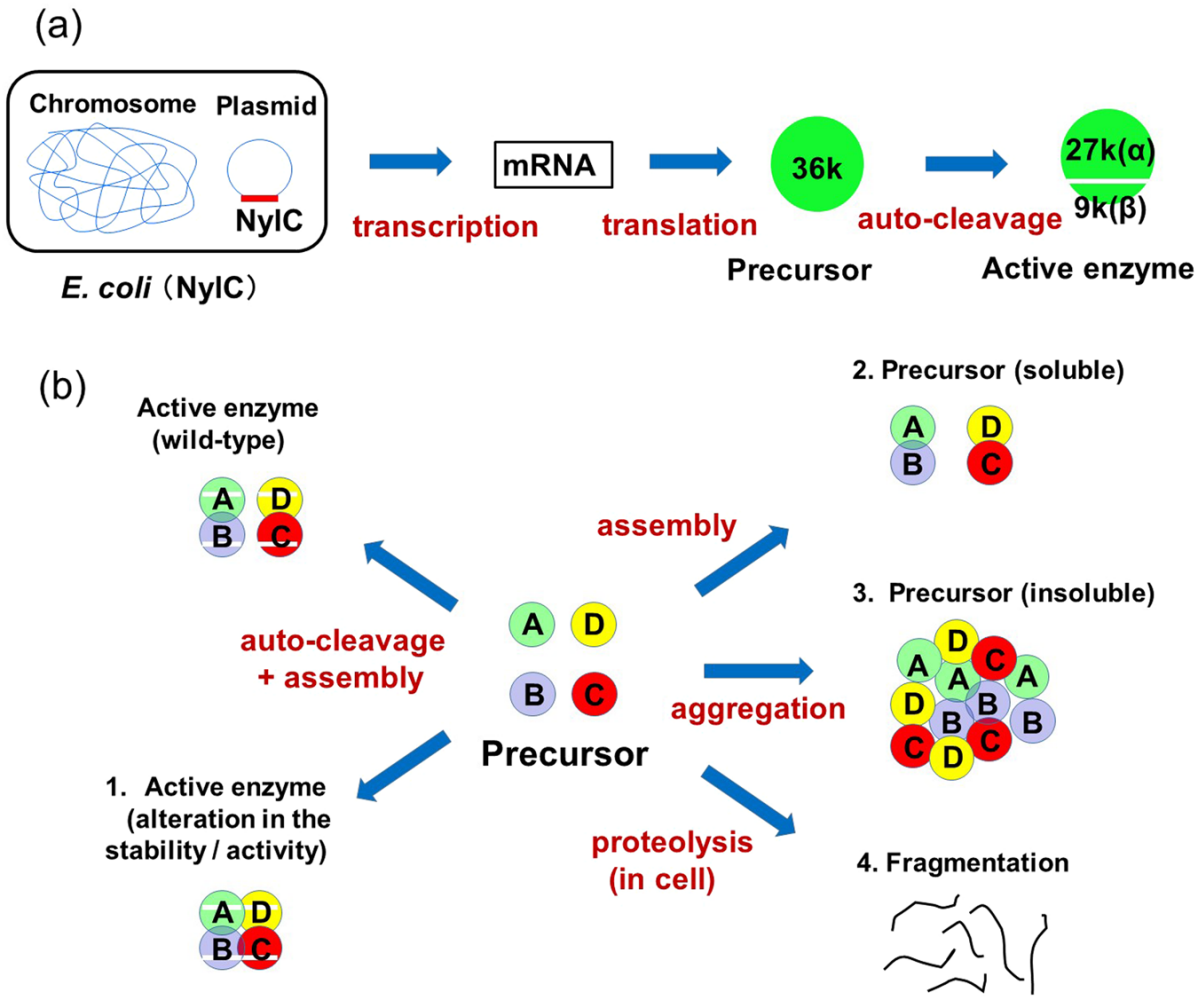
Genetic and mutational effects on the expression of the NylC. (**a**) The transcription, translation and autocleavage responsible for the expression of functional NylC are schematically shown. (**b**) Mutants obtained by PCR-induced random mutagenesis and site-directed mutagenesis were classified into four types. Type 1: obtained as active enzymes. Type 2: obtained as soluble precursors. Type 3: obtained as insoluble precursors (protein aggregation). Type 4: subjected to fragmentation during the cultivation/purification process (see Table 3).

Nylon hydrolase (NylC) is one of the three enzymes responsible for the endo-type degradation of the by-products of nylon-6 manufacture (cyclic and linear oligomers of 6-aminohexanoate (Ahx) with a degree of polymerization greater than three) and various aliphatic nylons (e.g., nylon-6 and nylon-66)^6–14^. NylC is a member of the N-terminal nucleophile (N-tn) hydrolase family^15–28^ and has been found in *Arthrobacter* (pOAD2 plasmid-encoded NylC; NylC_p2_), *Agromyces* (NylC_*A*_), and *Kocuria* (NylC_*K*_)^12–14^. NylC_*A*_ and NylC_*K*_ have 5 and 15 substitutions, respectively, in their sequences of 355 amino acid residues compared with the sequence of NylC_p2_ (Fig. S1), and both have 10–20 °C higher thermostability than does NylC_p2_^13^. The NylC enzymes are initially expressed as an inactive enzyme. However, the post-translational autocleavage of the nascent polypeptide between Asn266 and Thr267 generates an active enzyme with an α subunit (27 kDa) and a β subunit (9 kDa) (Figs 1 and 2)^7,8^. Based on the X-ray-crystallographic structure of NylC_*A*_, we reported that four αβ heterodimers (molecules A-D) form a doughnut-shaped quaternary structure^9^. In addition, we proposed that the nucleophilic N-terminal residue Thr267 in the β subunit functions as a catalytic residue for either autocleavage (from precursor to active enzyme) or substrate hydrolysis^9^. Moreover, the thermostability of NylC_p2_ (melting temperature (*T*_m_) identified by circular dichroism (CD) analysis = 52 °C) can be cumulatively enhanced by the D122G/H130Y/D36A/E263Q quadruple mutations (*T*_m_ = 88 °C) (Figs 1c, S1, and Table 1)^9^.

**Table 1:** Oligomeric states, thermostability and enzyme activity of wild-type NylC and mutant enzymes.

Enzyme	Amino acid substitutions in NylC_p2_ sequence	Oligomeric state*^1^	Subunit identified by SDS-PAGE	CD-melting analysis*^2^ *T*_m_ (°C)	Enzyme activity*^3^ (U mg^−1^)
NylC_p2_	—	Trimer/Dimer (Monomer)	α, β	52	7.1
NylC_*A*_	G111S D122G H130Y L137A V225M	Tetramer	α, β	60	15.7
NylC_*K*_	D36A A41V M50T I60V A62S G111S D122G H130Y L137A V225M T230G V231I V257L E263Q G354A	Tetramer (Monomer, Higher oligomer)	α, β	67	14.9
NylC_p2_-Y^130^	H130Y	Not tested	α, β	63	0.85
A^137^	L137A	Dimer (Monomer, Higher oligomer)	Precursor	41	< 0.1
G^122^Y^130^	D122G H130Y	Not tested	α, β	81	7.1
G^122^A^137^	D122G L137A	Dimer or monomer (Trimer)	Mixture of α, β, precursor	Not tested	Not tested
G^122^Y^130^A^36^Q^263^	D122G H130Y D36A E263Q	Tetramer (Monomer)	α, β	88	6.8

^*1^Oligomeric states of NylC in aqueous solution were estimated from analytical centrifugation. Minor populations are shown in parentheses. For example, in wild-type NylC_p2_, trimeric and dimeric αβ heterodimers were found at equal amounts, while a small population was present as monomeric αβ heterodimer. In NylC_p2_-A^137^, uncleaved dimeric molecules share the dominant population.

^*2^The thermostability determined previously by CD melting analysis (ref.^9^) is shown.

^*3^The enzyme activity was assayed at 30 °C under standard assay conditions (4 mg ml^−1^ of the cyclic Ahx oligomer) (ref.^9^).

In this study, we isolated various mutants that affect protein stability, autoprocessing of the precursor to the active enzyme, and subunit assembly. The mutants obtained were classified into the following four types (Fig. 2): active enzymes with altered thermostability or enzyme activity (type 1); soluble precursors (type 2); insoluble precursors (protein aggregation) (type 3); and fragments proteolyzed during the cultivation/purification process (type 4). Based on X-ray-crystallographic analysis, we describe the structural basis of the stability of nylon hydrolase and the fate of the protein, which is directed along one of the following pathways: i) correct subunit assembly generating dimer/tetramer structures, ii) inappropriate protein-protein interactions causing aggregation, or iii) intracellular disintegration of the nascent NylC polypeptide.

## Results and Discussion

### Oligomeric states of NylC

**Figure 3:**
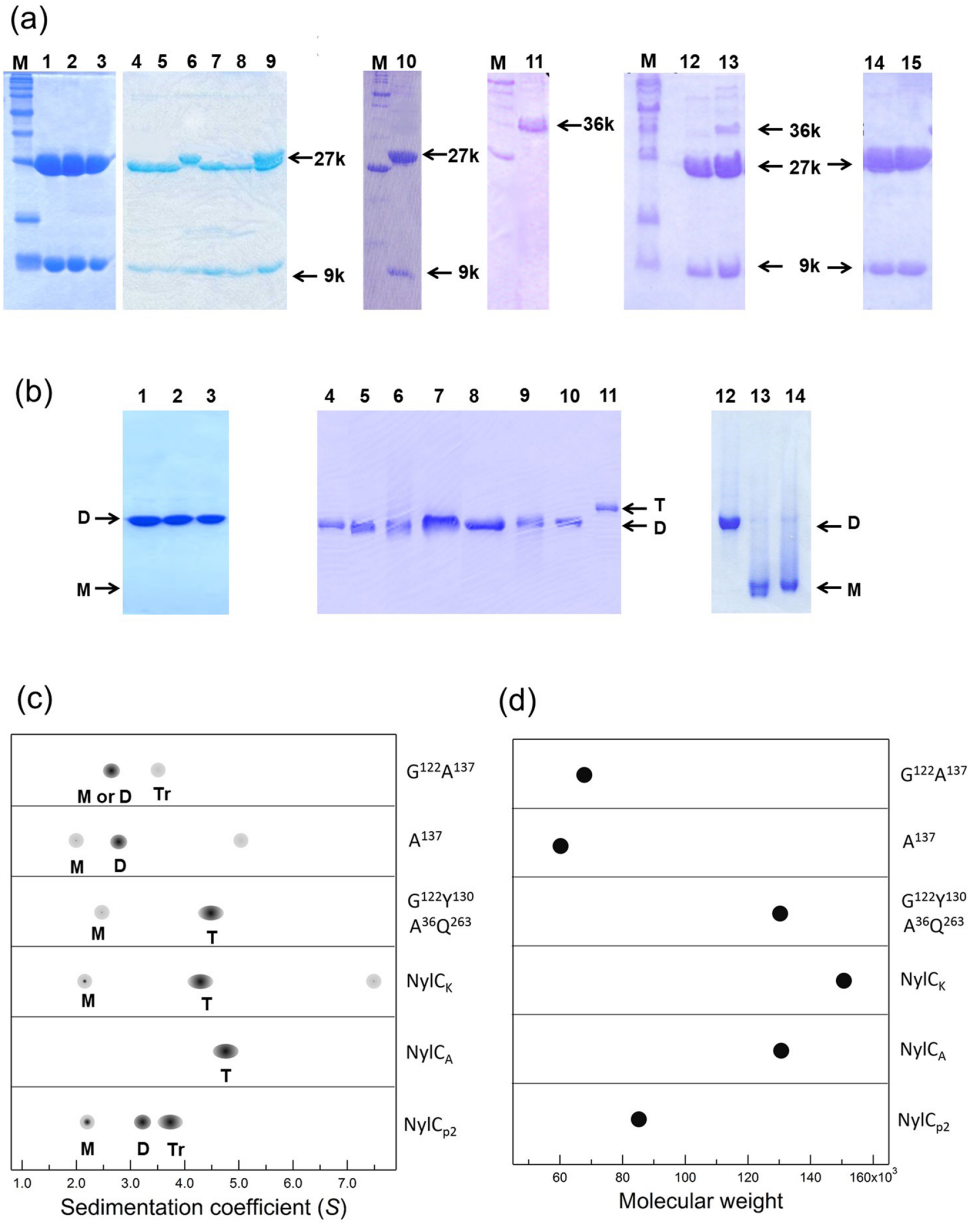
Polyacrylamide gel electrophoresis and ultracentrifugation analysis of wild-type and mutant enzymes. (**a**) SDS-PAGE (17.5% gel) of purified NylC. Lane M, molecular size marker; Lane 1, NylC_*A*_; Lane 2, NylC_*K*_; Lane 3, NylC_p2_; Lane 4, NylC_p2_-K^122^; Lane 5, NylC_p2_-L^122^; Lane 6, NylC_p2_-R^122^; Lane 7, NylC_p2_-V^122^; Lane 8, NylC_p2_-Q^122^; Lane 9, NylC_p2_-N^122^; Lane 10, NylC_p2_-G^122^Y^130^A^36^Q^263^; Lane 11, NylC_p2_-A^137^; Lane 12, NylC_p2_-G^122^Y^130^; Lane 13, NylC_p2_-G^122^A^137^; Lane 14, NylC_p2_-G^122^; and Lane 15, NylC_p2_-Y^130^. (**b**) Native-PAGE (10% gel) of purified NylC. The wild-type and most NylC mutants exhibited single bands corresponding to the dimeric structure (shown as “D”). However, some mutants (NylC_p2_-A^137^ and NylC_p2_-G^122^A^137^) exhibited a faster-migrating band (presumed to be the monomer and shown as “M”). In contrast, the most thermostable mutant, G^122^Y^130^A^36^Q^263^, showed the slowest-migrating band (presumed to be tetrameric and shown as “T”). Lane 1, NylC_*A*_; Lane 2, NylC_*K*_; Lane 3, NylC_p2_; Lane 4, NylC_p2_-L^122^; Lane 5, NylC_p2_-N^122^; Lane 6, NylC_p2_-Q^122^; Lane 7, NylC_p2_-R^122^; Lane 8, NylC_p2_-K^122^; Lane 9, NylC_p2_-V^122^; Lane 10, NylC_p2_-G^122^; Lane 11, NylC_p2_-G^122^Y^130^A^36^Q^263^; Lane 12, NylC_p2_-G^122^Y^130^; Lane 13, NylC_p2_-A^137^; and Lane 14, NylC_p2_-G^122^A^137^. The grouping of portions cropped from different gels was made explicit using delineation with dividing white space. Full-length gels are included in a Supplementary Information file (Fig. S8). (**c**,**d**) Ultracentrifugation analysis. The subunit assembly of the wild-type enzymes (NylC_p2_, NylC_*A*_, and NylC_*K*_) and mutant enzymes derived from NylC_p2_ (G^122^Y^130^A^36^Q^263^, A^137^, and G^122^A^137^) was analyzed by sedimentation velocity (**c**) and sedimentation equilibrium methods (**d**). The estimated oligomeric states, which are the monomer (M), dimer (D), trimer (Tr), and tetramer (T), for each peak in (**c**) are marked. The deviations of the *s* values of the same oligomeric state suggest that the shape of the protein structure (spatial location of loop regions and bulkiness of protein) affects the sedimentation coefficient in aqueous solution. The wild-type NylC_*A*_ gave a single peak (4.7 *S*) corresponding to a tetramer. The wild-type NylC_*K*_ gave one major peak (4.3 *S*) and two minor peaks (7.5 *S* and 2.1 *S*), suggesting that the monomer, tetramer, and higher oligomers coexisted in equilibrium. The wild-type NylC_p2_ gave two major peaks [3.7 *S* (trimer) and 3.2 *S* (dimer)] and a minor peak (2.1 *S*) (monomer). The G^122^Y^130^A^36^Q^263^ mutant gave one major peak corresponding to 4.5 *S* (tetramer) and a minor peak (2.4 *S*) (monomer). The A^137^ mutant gave a major peak (2.7 *S*) (dimer) and two minor peaks [2.0 *S* (monomer) and 5.0 *S*] molecular species. The G^122^A^137^ mutant gave a major peak of 2.6 *S* (monomer or dimer) and a minor peak of 3.5 *S* (trimer).

**Figure 4:**
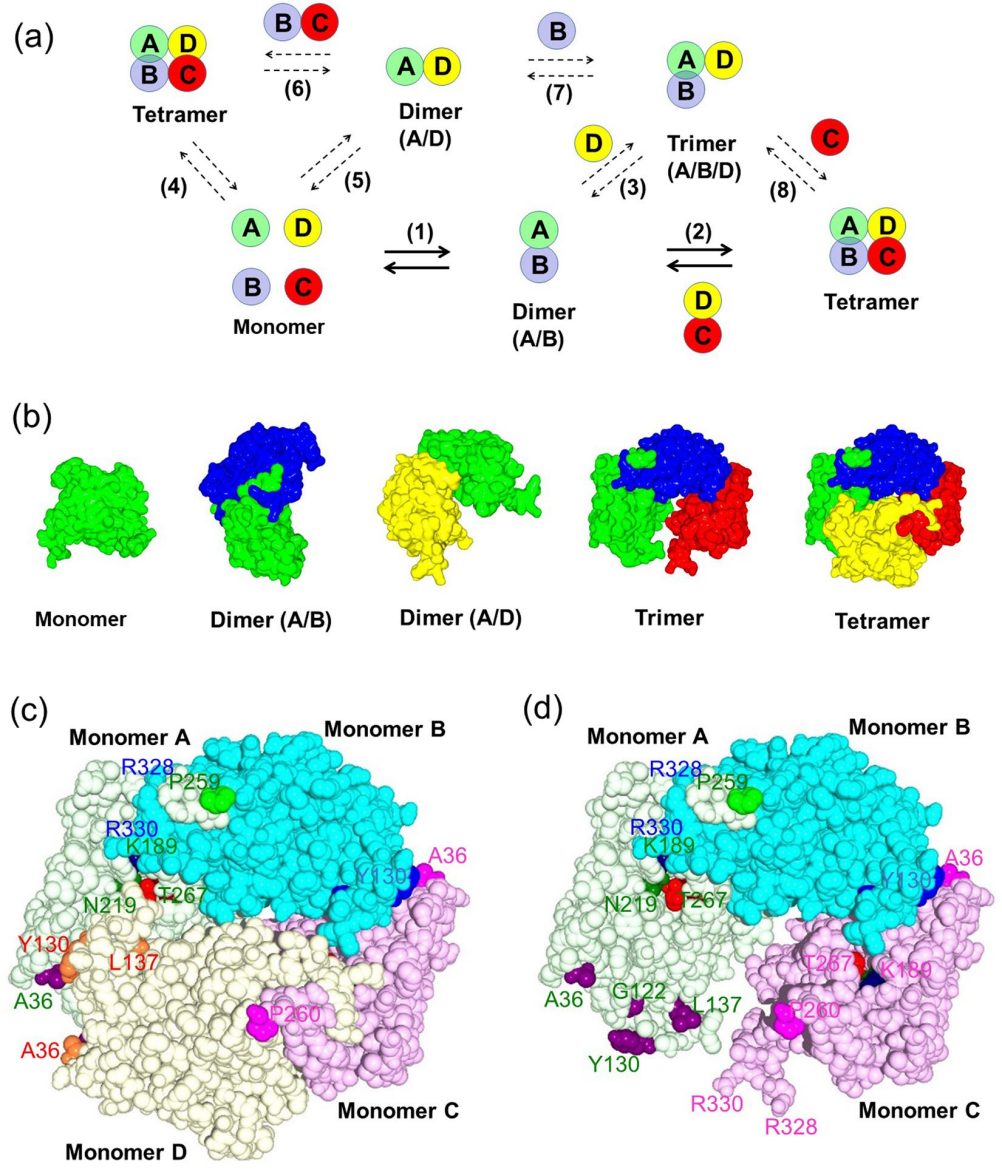
Subunit assembly and surface structure of NylC. (**a**) Model of the subunit assembly of NylC. The oligomeric states are dynamically altered depending on the structural changes caused by mutations. Typical association/dissociation reactions (reactions 1–8) are shown. Major assembly and dissociation reactions are assumed to be in equilibrium and are shown as solid lines (reactions 1 and 2), while the alternative equilibria shown as dashed lines may be possible. To simplify the designation of each monomer molecule in the quaternary structure, both the uncleaved precursor and the cleaved enzyme (αβ heterodimer) are expressed as molecules A-D. (**b**) Monomer (A), dimer (A/B and A/D), trimer (A/B/C), and tetramer structures are shown as surface models. Contacts at the A/B and C/D interfaces (3,121 Å^2^) were observed to be more extensive than those at the B/C and A/D interfaces (1,451 Å^2^). (**c**) The tetramer structure of the NylC_p2_-G^122^Y^130^A^36^Q^263^ mutant is shown as a *space-filling* model. The overall structures of monomer molecules A, B, C, and D are shown in *light green*, *light blue*, *light pink*, and *light yellow*, respectively. To highlight the catalytic residues and mutated sites, the catalytic nucleophile Thr267 (in the N-terminus of the β subunit in monomer A) and other presumed catalytic residues (Lys189 and Asn219) are shown in *red*, *dark blue*, and *dark green*, respectively. The mutated residues (Ala36, Gly122, Tyr130, and Leu137) in monomer A are shown in *purple*. The mutated residues (Ala36, Tyr130, and Leu137) in monomer D are shown in *orange*. The conformation of the Glu263 side chain in NylC_p2_-G^122^Y^130^A^36^Q^263^ could not be determined due to poor electron density. Pro259/Pro260, which is linked to the loop 5 region, is shown in *green* (monomer A) and *magenta* (monomer C). Arg328 and Arg330 in loop 7 are marked. (**d**) To show the structure of the A/D interface in the NylC_p2_-G^122^Y^130^A^36^Q^263^ mutant, the structure (*space-filling model*) is shown by omitting monomer D.

To study the oligomeric states of nylon hydrolase, we purified three wild-type NylCs (NylC_p2_, NylC_*A*_, and NylC_*K*_) and several NylC mutants to homogeneity and analyzed the differences in the patterns of sedimentation using analytical ultracentrifugation (Fig. 3). As described below, the assembly of monomeric units (A-D) dynamically switched between the monomer, dimer (A/B or A/D), trimer, and tetramer (as well as higher oligomers), and the oligomeric states were affected by mutations, especially at the subunit interface (Fig. 4). In addition, amino acid substitutions at specific positions altered not only the protein stability but also the catalytic activity, as well as the processability to the active enzyme (Table 1).

Wild-type NylC_*A*_ produced a single peak in the sedimentation velocity [sedimentation coefficient at 20 °C (*s*_20_) = 4.7 *S*], indicating that the enzyme exhibited a narrow distribution in the oligomeric states (Fig. 3c). The average molecular weight (M_AV_) of NylC_*A*_ was 130,928 based on sedimentation equilibrium analysis (Fig. 3d). Therefore, NylC_*A*_ was mainly present as a tetramer of αβ-heterodimers in aqueous solutions (Table 1). In contrast, the M_AV_ of wild-type NylC_p2_ was 85,602 based on sedimentation equilibrium analysis. This value was in good agreement with the molecular weight (93,000) estimated by gel-filtration chromatography^11^. These results suggest that the trimeric structure of αβ-heterodimers (3.7 *S*), as well as the monomeric (2.1 *S*) and dimeric (3.2 *S*) structures, was formed in the case of NylC_p2_. Wild-type NylC_*K*_ and the most thermostable mutant, NylC_p2_-G^122^Y^130^A^36^Q^263^ (*T*_m_ = 88 °C), should exist mainly as a tetramer of αβ-heterodimers (M_AV_ = 151,384 and 130,895, respectively) (Fig. 3d).

The *M*_AV_ values of NylC_p2_-A^137^(60,258) and NylC_p2_-G^122^A^137^(67,397) obtained by equilibrium analysis were between the values corresponding to the monomer (36 kDa) and the dimer (72 kDa). The presence of the monomeric unit was also confirmed by native polyacrylamide gel electrophoresis (PAGE) analysis of the NylC_p2_-A^137^ and NylC_p2_-G^122^A^137^ mutants (Fig. 3b). SDS-PAGE analysis revealed that the NylC_p2_-A^137^ mutant was obtained as a precursor (type 2 mutation), whereas the more stable G^122^A^137^ mutant was obtained as a mixture of the precursor and the α- and β-subunits (Fig. 3a, lanes 11 and 13).

The oligomeric states of NylC enzymes should also depend on the enzyme concentration and environmental conditions to which the enzymes are exposed. Under the enzyme concentrations (0.34 mg ml^−1^) used for ultracentrifugation analysis, NylC_p2_ was in a monomer/dimer/trimer equilibrium, NylC_*A*_ provided a homogeneous tetrameric structure, but NylC_*K*_ produced higher oligomer and monomer molecules in addition to the major tetrameric structures (Fig. 3). In contrast, native PAGE analysis of wild-type NylC_p2_, NylC_*A*_, NylC_*K*_, and most mutants yielded a single band, presumed to be a dimer (Fig. 3b, lanes 1–3). These results suggest that the oligomeric states are more homogeneous in the gel matrix of polyacrylamide and that the tetrameric (NylC_*A*_ and NylC_*K*_) and trimeric (NylC_p2_) molecules are likely to dissociate into dimeric structures.

The expected oligomeric states in aqueous solution, processability from the precursor to the active enzyme, thermostability, and enzyme activity obtained in the present experiments are summarized in Table 1. In this study, we demonstrate that the local alterations induced by mutations in certain cases generated additional contacts with the adjacent monomers, resulting in protein aggregation (type 3 mutation in Fig. 2). In contrast, if the structural alterations induced by mutations weakened the interactions required for protein assembly, the nascent monomeric polypeptides did not always fold and assemble to form stable oligomeric structures (type 4 mutation). The molecular basis of subunit assembly is discussed below on the basis of the three-dimensional structure (Fig. 4).

### Mutations that affect the protein stability and autoprocessing

Among the various mutants obtained from wild-type NylC_p2_, which differ in thermostability, a single D122G mutation drastically improves the thermostability by 24 °C^9^. To extensively analyze the effect of amino acid substitution at position 122, we constructed 10 new mutants in which Asp122 of NylC_p2_ was replaced with other amino acids by site-directed mutagenesis. The wild-type and mutant enzymes exhibited the typical far-UV CD spectrum (200–250 nm) for non-denatured proteins at 25 °C, whereas for CD analysis at 95 °C, the enzymes exhibited the typical pattern for denatured proteins (Fig. S2). The values of *T*_m_ (melting point of the heat denaturation) and Δ*H* (change in enthalpy for the global unfolding of proteins) were determined by regression analysis using nonlinear least-squares fitting of the CD melting curve (Fig. S2c). The *T*_m_ values increased in the order of Asp122 (wild-type NylC_p2_) < Leu < Gln < Asn < Arg < Lys < Val < Gly and ranged from 52.9 °C to 75.1 °C (type 1 mutation in Fig. 2) (Table 2). The CD analyses were performed under more diluted enzyme concentrations (0.1 mg ml^−1^) than the ultracentrifugation analysis. However, the tendency of the thermostability obtained by the residual enzyme assay using a higher concentration (1 mg ml^−1^) was largely similar to the stability obtained by the CD analysis (Table 2 and Fig. S3). Therefore, we estimated the molecular basis underlying the protein stability on the basis of the structure (X-ray crystallography) and the oligomeric states (ultracentrifugation and native PAGE analysis).

**Table 2:** Thermostability and enzyme activity of NylC mutant enzymes.

Enzyme	Amino acid substitutions in NylC_p2_ sequence	Subunit identified by SDS-PAGE	CD-melting analysis*^1^		Thermostability*^2^ (°C) by residual activity assay	Enzyme Activity*^3^ (U mg^−1^)
			*T*_m_ (°C)	∆*H* (kJ mol^−1^)		
NylC_p2_	—	α, β	52.9 ± 0.1	−401 ± 15	42	7.1
NylC_p2_-G^122^	D122G	α, β	75.1 ± 0.1	−543 ± 12	73	6.3
V^122^	D122V	α, β	74.7 ± 0.0	−655 ± 14	72	6.5
K^122^	D122K	α, β	70.8 ± 0.1	−520 ± 13	65	7.5
R^122^	D122R	α, β	69.5 ± 0.1	−409 ± 8	65	7.1
Q^122^	D122Q	α, β	64.3 ± 0.1	−252 ± 4	61	6.1
N^122^	D122N	α, β	66.7 ± 0.1	−394 ± 11	60	7.5
L^122^	D122L	α, β	60.2 ± 0.1	−355 ± 7	55	5.7
H^122^	D122H	Enzyme is not expressed in cell.				
P^122^	D122P					
W^122^	D122W					

^*1^*T*_m_ (melting point of the heat denaturation) and Δ*H* (change in enthalpy for the global unfolding of proteins) were determined from the CD melting curve by regression analysis using nonlinear least squares fitting.

^*2^Enzyme solutions were incubated at various temperatures for 30 min, and the residual enzyme activity was analyzed (Fig. S3).

^*3^The enzyme activity was assayed at 30 °C under standard assay conditions (4 mg ml^−1^ of the cyclic Ahx oligomer) (ref.^9^).

In the H^122^, W^122^, and P^122^ mutants of NylC_p2_, no protein bands corresponding to the active enzyme (27 kDa and 9 kDa) or the precursor (36 kDa) were detected by Western blot analysis, whereas low levels of NylC-antigenic protein were detected by a direct ELISA (Table 3 and Fig. S4). Our attempts to express mutant enzymes having a single D122H, D122W, or D122P substitution in NylC_p2_ were unsuccessful, even though we conducted three independent experiments. The elution profiles from ion-exchange column chromatography of the cell extracts of the *E*. *coli* clone showed no protein peaks corresponding to the NylC fractions (Fig. S5). We speculate that protein destabilization results in the denaturation of the protein even at the cultivation temperature. Consequently, the nascent mutant NylC polypeptide is cleaved into fragments by intracellular proteases (type 4 mutation in Fig. 2).

**Table 3:** Immunological detection of NylC-antigenic protein and RT-PCR analysis.

Mutant enzymes derived from NylC_p2_	Mutant phenotype*^1^	Western blotting*^2^		Direct ELISA*^3^ (µg ml^−1^)	RT-PCR*^4^ (%)
		Soluble	Insoluble		
P^122^	Type 4	—	—	8.2	129
H^122^	Type 4	—	—	2.4	142
W^122^	Type 4	—	—	3.2	112
G^122^Y^130^A^36^Q^263^	Type 1	27 kDa, 9 kDa	—	11.4	100
G^122^Y^130^A^36^Q^263^-T^179^	Type 4	—	—	8.1	121
G^122^Y^130^A^36^Q^263^-T^295^	Type 1	27 kDa, 9 kDa	—	17.7	113
G^122^Y^130^A^36^Q^263^-D^299^	Type 4	—	—	4.4	154
G^122^Y^130^A^36^Q^263^-E^299^	Type 3	—	36 kDa	11.7	106
G^122^Y^130^A^36^Q^263^-V^75^	Type 3	—	36 kDa	1.1	120
G^122^Y^130^A^36^Q^263^-R^106^	Type 3	—	36 kDa	0.2	118
G^122^Y^130^A^36^Q^263^-L^141^	Type 4	—	—	2.9	124
G^122^Y^130^A^36^Q^263^-L^157^	Type 3	—	36 kDa	3.1	96
G^122^Y^130^A^36^Q^263^-N^191^	Type 1	27 kDa, 9 kDa		14.3	121
G^122^Y^130^A^36^Q^263^-D^235^	Type 3	—	36 kDa	6.9	67

^*1^NylC mutants were classified into four types (see Fig. 2b). Type 1: obtained as active enzymes, Type 2: obtained as soluble precursors (e.g., NylC_p2_-A^137^; see Table 1). Type 3: obtained as insoluble precursors (protein aggregation). Type 4: subjected to fragmentation during the cultivation/purification process.

^*2^The cell extract (soluble fraction) and precipitates obtained by the centrifugation of sonicated cells (insoluble fraction) were boiled in SDS, and NylC-antigenic proteins were detected by Western blot analysis using anti-NylC antibody (Fig. S4).

^*3^The amount of NylC-antigenic protein was analyzed by direct ELISA using anti-NylC antibody. The antigenic protein was estimated by subtracting the background level (4.9 µg ml^−1^ for cell extracts harboring the vector pBluescript) from the data obtained for *E*. *coli* clones harboring the mutant *nylC* gene.

^*4^To check the expression of the mutant *nylC* gene, we prepared RNA samples from *E*. *coli* clones. Reverse transcription (RT)-PCR analysis revealed that PCR products corresponding to *nylC*-mRNA were present in all mutants.

### X-ray-crystallographic analysis of wild-type NylC_p2_ and typical mutant enzymes

The crystal of NylC_*A*_ contains fifteen monomeric molecules in a single unit cell^9^. Such a large number of monomers in one unit cell is not suitable for structural analysis at high resolution. Therefore, we screened and established a new crystallization condition, which generated crystals with only two molecules (corresponding to the A/B dimer) related by a non-crystallographic two-fold axis in an asymmetric unit (space group *C*222_1_)^11^. We performed X-ray-crystallographic studies of wild-type NylC_p2_ and the seven typical mutants at 1.05–2.00 Å resolution (Table S1). The four identical αβ heterodimers (molecules A-D) were mutually related by D2 symmetry (Fig. 1b). Each monomer molecule (A-D) of NylC contained five α-helices (α1–α5), five 3_10_-helices (η1–η5), 20 β-strands (β1–β20), and 29 loop regions (Fig. 1d), generating a stacked αββα core structure (Fig. 1e). The overall structure of NylC_p2_ was almost identical to that of NylC_*A*_ determined previously^9^ (Fig. S6) with a root mean square deviation (rmsd) of 0.552 Å. The rmsd of the main-chain atoms between NylC_p2_ and NylC_*A*_ and between NylC_p2_ and NylC_p2_-G^122^Y^130^A^36^Q^263^ was generally less than 1 Å, but loop 1 (positions 15–34), loop 2 (positions 88–93), loop 3 (positions 125–133), α3 (positions 246–257), loop 5 (positions 258–269), and loop 7 (positions 319–333) displayed significant structural differences (Fig. S6). It should be noted that α1 and its adjacent loop 3 regions showed the largest structural difference between NylC_*A*_ and NylC_p2_, whereas a difference of less than 2 Å was found between NylC_*A*_ and NylC_p2_-G^122^Y^130^A^36^Q^263^ (Fig. S6b). The structural differences in the α1 and loop 3 regions that included position 122 seemed to correlate with the thermostability of the enzymes (see below). Large deviations around position 30 were due to the difference in crystal contacts. The loop 5 region, which was close to the autocleavage site, displayed high temperature factors, and consequently, the residues at positions 261–266 were invisible (Fig. S7).

Three amino acid residues corresponding to the substitutions D122G, H130Y, and D36A (increasing the protein stability of NylC_p2_) and one amino acid residue corresponding to the substitution L137A (decreasing the protein stability) were located at the A/D interface, and the glutamate residue corresponding to the substitution E263Q was found at the A/B interface (Figs 1 and 4). Since there were no detectable contacts between monomers A and C, we focused on the effects of amino acid substitutions on the protein stability and structural alterations between monomers A, B, and D on the basis of the structure of monomer A.

### Interaction between helices and the partial asymmetry caused by the mutations

**Figure 5:**
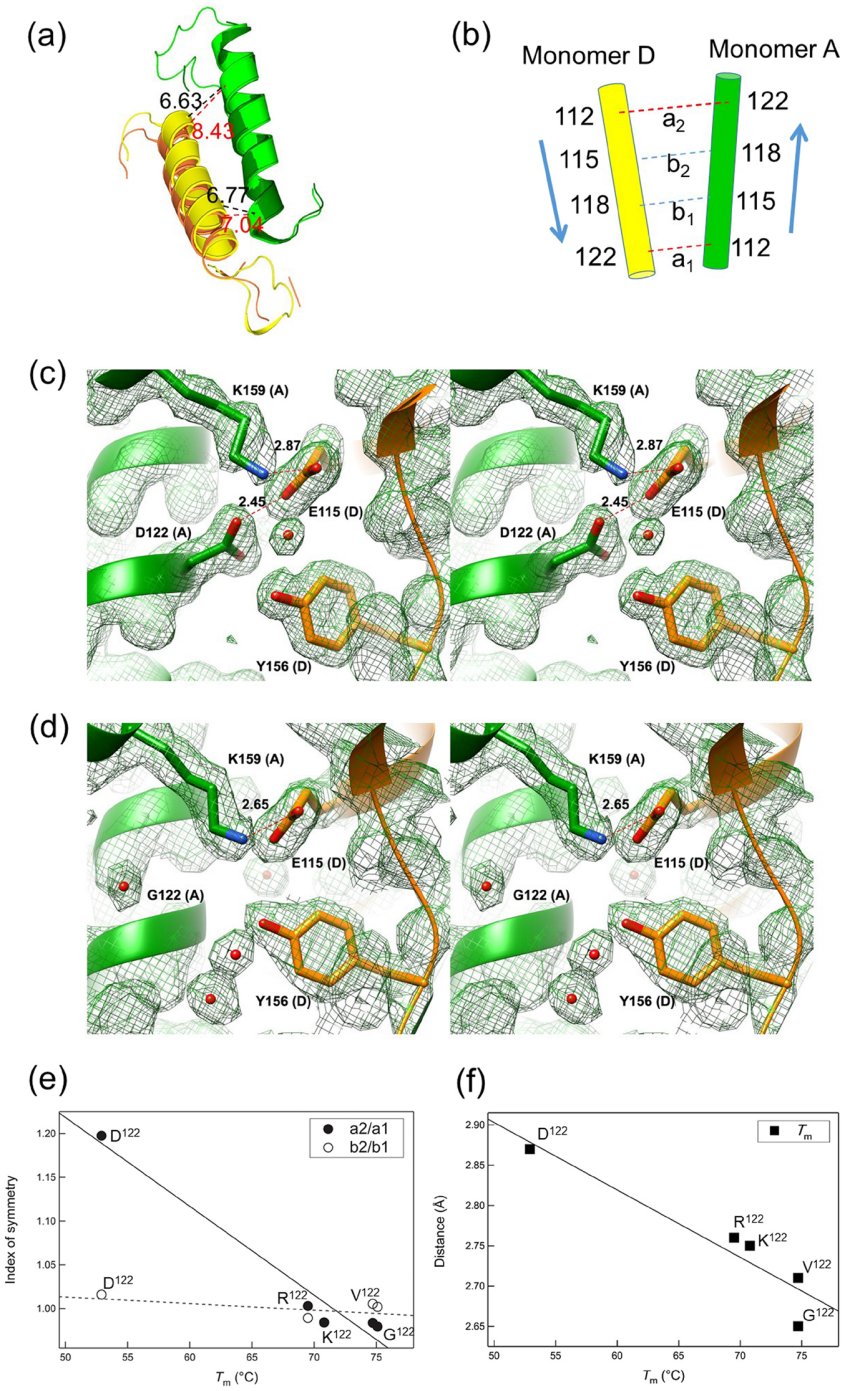
Subunit interactions between monomers A and D. (**a**) The structure of helix α1 and its adjacent loop regions in monomers A and D is shown as a *ribbon diagram*. The structure of NylC_p2_-G^122^ (monomer A, *green*; monomer D, *yellow*) is superimposed on the structure of NylC_p2_ (monomer A, *dark green*; monomer D, *orange*). (**b**) The relationship between the α1 helices in monomers A and D is illustrated. a_1_, distance between position 112 (monomer A) and position 122 (monomer D) at Cα; a_2_, distance between position 122 (monomer A) and position 112 (monomer D); b_1_, distance between position 115 (monomer A) and position 118 (monomer D) at Cα; b_2_, distance between position 118 (monomer A) and position 115 (monomer D). (**c**,**d**). The structure of NylC_p2_ (**c**) and the NylC_p2_-G^122^ mutant (**d**) at the A/D monomer interface is shown as a stereo diagram. The side chains of Asp122, Gly122, and Lys159 (monomer A), as well as Glu115 and Tyr156 (monomer D), are shown as stick diagrams in 2*F*_o_-*F*_c_ electron density maps. The contour level of the electron density map is 1σ. Possible hydrogen bonds and contacts between two atoms are indicated as dotted lines with the distances listed in Å. (**e**) The index of symmetry (a_2_/a_1_ and b_2_/b_1_) is plotted against the protein stability (melting temperature: *T*_m_) estimated from CD analysis of NylC_p2_ (wild-type) and NylC_p2_-R^122^, K^122^, V^122^, and G^122^-mutants. (**f**) The distance from Lys159-NH_3_^+^ (located on helix α2 in monomer A) to Glu115-Oε_1_^−^ (located on helix α1 in monomer D) is plotted against the *T*_m_.

At the A/D interface, helix α1 of monomer A was juxtaposed next to helix α1 of monomer D (Fig. 1b). However, the D2 symmetry was partly altered by mutations at position 122. For example, in wild-type NylC_p2_ (Asp122, *T*_m_ = 52.9 °C), the distance between position 112 (monomer A) and position 122 (monomer D) at Cα (designated as “a_1_”) was 7.04 Å (Fig. 5 and Table S2). In contrast, the distance between position 122 (monomer A) and position 112 (monomer D) (designated as “a_2_”) was considerably greater with a value of 8.43 Å. Plotting the protein stability (*T*_m_ value) estimated from the CD analyses against the distance between the two monomer molecules revealed that the protein stability exhibited an inverse correlation with the a_2_/a_1_ ratio (index of symmetry). In other words, the ratio changed from 0.979 to 1.197 from the wild-type (Asp122) to the mutant (Arg122, Lys122, Val122, and Gly122) enzyme, although this value should be equal to one for a perfectly symmetric structure (Fig. 5e). From the distance between positions 115 and 118 at the Cα of the α1 helices of the monomers A and D (designated “b_1_” and “b_2_”, respectively), the b_2_/b_1_ ratio was similarly calculated. Since the values (b_2_/b_1_ = 0.984–1.016) were closer to the theoretical value, the structural symmetry was better conserved at position 115/118. Thus, the partial asymmetry at the A/D interface inhibits tetramer formation but generates an irregular trimeric structure in aqueous solution, as shown in the proposed model (Fig. 4).

A clearer correlation was found between thermostability and the nearest distance between the side-chain atoms on helix α2 and helix α1. In wild-type NylC_p2_, Lys159-NH_3_^+^ (located on helix α2 in molecule A) was 2.87 Å away from Glu115-Oε_1_^−^ (located on helix α1 in molecule D) (Fig. 5c and Table S3). In contrast, the distance was reduced to 2.65 Å in the thermostable Gly122 enzyme (Fig. 5d). Notably, this distance decreased with an almost linear correlation with increasing thermostability in the wild-type and mutant enzymes (Fig. 5f). The side-chain atoms of Lys122 existed as two conformers, with the distance of one conformer showing a linear relationship with thermostability. We suggest that the electrostatic effect between the two residues will enhance the subunit binding around Gly122 of helix α1. However, in NylC_p2_, the amino acid residue at position 122 was replaced with Asp, and the close proximity of the acidic residue Asp122 (close to Glu115) reduced this stabilization effect. Since the correlation between the Lys159-Glu115 distances and the enthalpy change (Δ*H*) for the unfolding of proteins was not strong (Table 2), the overall stabilization effects should be determined by the additional stabilization effect, probably by the interaction at the loop regions as described below.

In the case of NylC_p2_ mutants having single Asn^122^ (*T*_m_ = 66.7 °C), Gln^122^ (*T*_m_ = 64.3 °C), and Leu^122^ mutations (*T*_m_ = 60.2 °C), crystals were not obtained even under the crystallization conditions optimized for NylC_p2_. Therefore, it is likely that the local structural changes caused by the mutations altered the precise orientation of the enzyme molecules required for crystal formation.

### Stabilization of loop regions by subunit interactions

**Figure 6:**
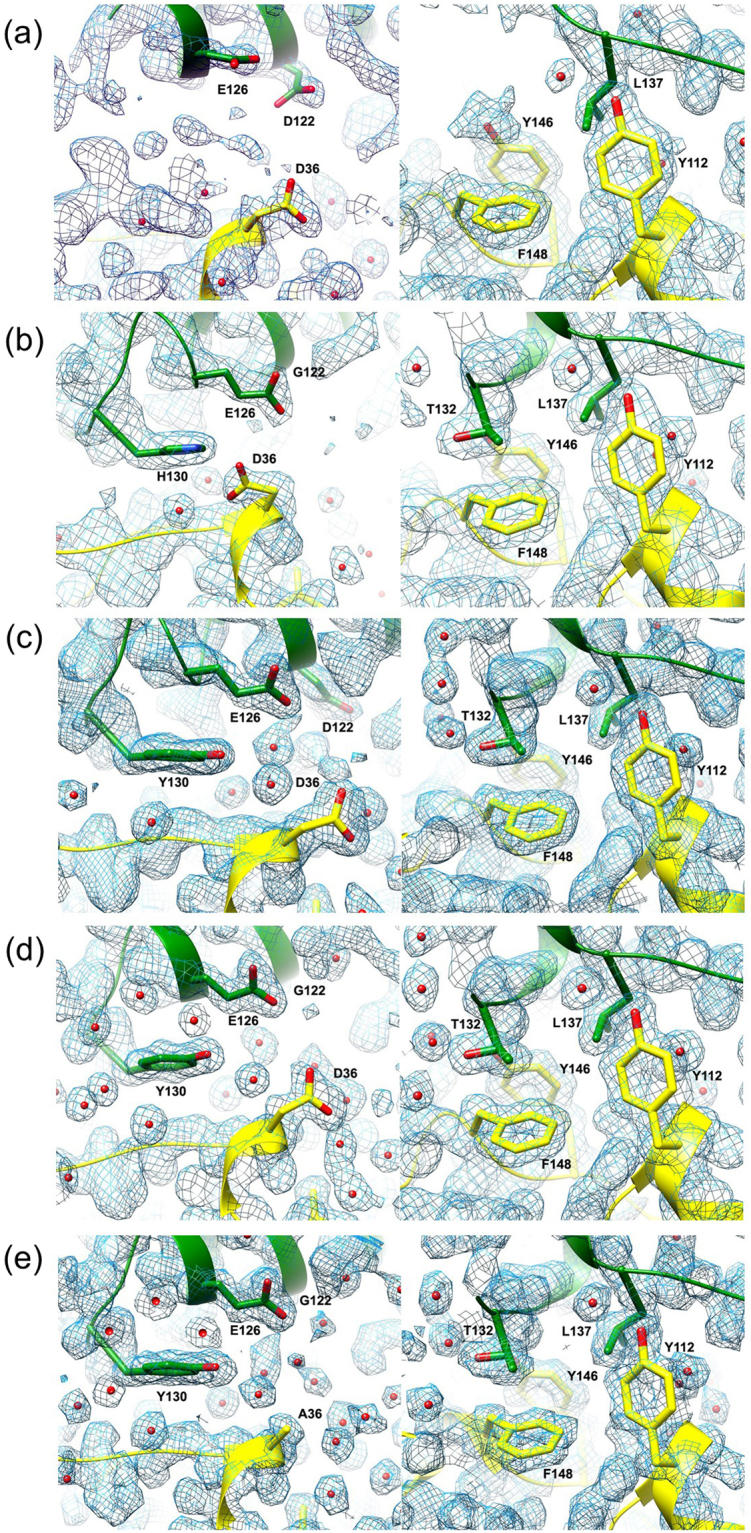
Structure of loop regions located at the A/D monomer interface and catalytic centers. The 2*F*_o_-*F*_c_ electron density maps of NylC_p2_ (**a**), NylC_p2_-G^122^ (**b**), NylC_p2_-Y^130^ (**c**), NylC_p2_-G^122^Y^130^ (**d**), and NylC_p2_-G^122^Y^130^A^36^Q^263^ (**e**) are shown. Left panel: amino acid residues in monomer A (Asp/Gly122, Glu126, and His/Tyr130) and monomer D (Asp/Ala36) are shown as *stick models*. Right panel: amino acid residues in monomer A (Thr132 and Leu137) and monomer D (Tyr112, Tyr146, and Phe148) are shown as *stick models*. In the thermostable enzymes (**c–e**), “loop 3” and α3 (containing Tyr130, Thr132, and Leu137) (in monomer A) are mutually stabilized by contacts with “loop 1” and “loop 4” (containing Tyr146 and Phe148) in monomer D. Leu137 (in monomer A) has contacts with monomer D at Tyr146, Phe148, and Tyr112 (in helix α1), suggesting that hydrophobic interactions around Leu137 stabilize the protein structure. D36A substitution in NylC_p2_-G^122^Y^130^Q^263^ mutant (*T*_m_ = 84 °C) contributes to the increased stability, probably by the reduction of electrostatic repulsion between Asp36-COO^−^ (monomer D) and Glu126-COO^−^ (monomer A) (**e**).

His130 and Leu137 in NylC_p2_ were located in a loop between helix α1 and β-strand β7 (designated “loop 3”) (Fig. 1d). However, the structures of loop 1 and loop 3 containing His130 in NylC_p2_ were not built during X-ray-crystallographic analysis due to the poor electron density (Fig. 6a). Moreover, the temperature factors (index of thermal motion in a crystallographic structure) of loop regions 1–3 in NylC_p2_ were significantly larger than those of the corresponding regions in the thermostable NylC-G^122^Y^130^ mutant (Fig. S7). In the thermostable enzymes, loop 3 and η3 (in monomer A) was mutually stabilized by contacts with loop 1 and loop 4 in monomer D (Fig. 6). Moreover, a single L137A substitution in the η3 region of the native NylC_p2_ resulted in a decrease in thermostability by 11 °C. The L137A substitution resulted in a loss of the autocleavage function, which is required for converting the precursor to the active enzyme. The effect of this amino acid substitution on autocleavage is discussed below.

The C-terminal region of the α subunit, including helix α3 and Pro260, flipped out toward the adjacent monomer at each subunit interface (Fig. 4c and d). Glu263 was located in the terminal region (loop 5) of the α subunit, which had a poor electron density distribution in the X-ray diffraction analysis of the wild-type and all NylC mutants. The Asp319-Val333 region (loop 7) was also flipped out towards the adjacent monomer. Thus, the loop 5 of monomer A was co-located with the loop 7 of monomer B at the surface of the tetramer structure (Fig. 4c and d). In the G^122^Y^130^A^36^Q^263^ mutant, the acidic amino acid residue Glu263 was replaced with a neutral Gln, suggesting that the alterations in the electrostatic environment induced by the E263Q mutation contributed to improving the subunit interactions. We confirmed that the E263Q substitution in G^122^Y^130^A^36^ (*T*_m_ = 84 °C) increased thermostability by 4 °C^9^.

We found that the V225M substitution, which was close to Gln299, decreased the expression and/or stability of NylC_p2_ in cells, whereas this mutation increased the stability of NylC-G^122^Y^130^ by 2 °C^9^. Therefore, the total stabilization effects depended on the combined interactions with the surrounding residues. Since Gln299 (in monomer A) was close to Arg296 (in monomer B), the replacement of the neutral amino acid Gln299 with acidic amino acids (Glu299 or Asp299) was expected to enhance the electrostatic interaction with the positively charged guanidium group of Arg296 at the A/B monomer interface. However, we found that the mutant enzyme with the Q299E substitution was aggregated (type 3 mutation), while the Q299D mutant enzyme was degraded at the cultivation stage (type 4 mutation) (Fig. 2b).

### Cumulative mutational effects directing the successive fates of the enzyme

To analyze successive mutational effects on the most thermostable enzyme G^122^Y^130^A^36^Q^263^, we constructed various mutants by PCR-induced random mutagenesis. Of the 100 mutants isolated after random mutagenesis using the G^122^Y^130^A^36^Q^263^ enzyme, eight mutant enzymes with a single mutation were selected, and their properties were examined (Table 3). Only two mutants (H295T, D191N) were similar to the parental G^122^Y^130^A^36^Q^263^ mutant in the immunological analysis. However, the successive mutations caused protein aggregation (type 3 mutation) or intracellular degradation of the precursors in the cells (type 4 mutation), as described below.

#### Mutations causing protein aggregation

Western blot analysis of the NylC_p2_-G^122^Y^130^A^36^Q^263^-Glu299 mutant using a polyclonal NylC antibody and *E*. *coli* cells lysed by sonication was performed. The precursor (36 kDa) band was detected in the insoluble fraction, but no NylC-antigenic protein band was detected in the soluble fraction (Table 3 and Fig. S4). In addition, mutant enzymes with the I75V, L106R, P157L, or N235D single substitutions obtained by PCR-induced random mutagenesis exhibited a similar pattern in Western blot analysis (Table 3 and Fig. S4). Glu299 was located at the A/B monomer interface and on β-strand β19 in monomer A (Fig. 1). Asn235 was located at the A/B interface, which was close to Q299. In contrast, two mutations (I75V and L106R) were located on the central β-sheets and loop region, which were close to helix α1. Another mutation (P157L) was located on loop 4, which was also close to helix α2 (Fig. 1). Thus, the aggregation of the enzyme and the inability to autocleave the precursor were caused by mutations at various sites, rather than specific positions in the monomer structure.

#### Mutations causing intracellular degradation of the enzyme

The Asp299-mutant enzyme was thought to be degraded by intracellular proteases during cultivation of the *E*. *coli* clone, since the results obtained by immunological detection (Western blot analysis and sandwich ELISA) and RT-PCR analysis were very similar to the results for the NylC_p2_-His122, -Pro122, and -Trp122 enzymes (Table 2). The S141L or A179T substitutions in NylC-G^122^Y^130^A^36^Q^263^ caused similar phenotypic changes. S141 was located on β-strand β7 (Fig. 1). S141 was located close to A137, and the L137A substitution in NylC_p2_ showed pleiotropic effects on subunit assembly, autoprocessing, and thermostability (described below).

As stated above, amino acid substitution at a specific position (e.g., position 122 at the A/D interface and position 299 at the A/B interface) altered the local structure of the surrounding residues. Therefore, it was likely that these local structural alterations led to additional contacts with the adjacent monomers. We hypothesize that many monomer molecules were associated in an inappropriate orientation of monomers through these additional contacts, resulting in protein aggregation (type 3 phenotype). In contrast, if the structural alterations induced by mutations weakened the significant interactions required for protein assembly, the nascent unfolded monomeric polypeptides would be subjected to degradation by intracellular proteases (type 4 phenotype).

### Effects of mutations on autocleavage

From the three-dimensional structure of NylC_*A*_ and the results obtained from the other N-tn family enzymes^15–28^, we proposed that catalytic residue Thr267 is responsible for the autocleavage of the precursor and substrate hydrolysis^9^. We proposed that the auto-processing reaction is initiated by the nucleophilic attack of Thr267-O_γ_ on Asn266-Cα carbon, generating a tetrahedral intermediate. This intermediate rearranges into an ester intermediate (N-O acyl shift) that is subsequently hydrolyzed by an adjacent water molecule, producing the active enzyme^9^. The NylC_p2_-A^137^ mutant (type 2 mutant) was obtained as a precursor, whereas the more stable G^122^A^137^ mutant was obtained as a mixture of the precursor and α- and β-subunits (Fig. 3a, lanes 11 and 13). In the three-dimensional model, the α3 region (in monomer D) containing Phe134 and Leu137 is spatially close to Thr267 (processing site). The location of these structures around the catalytic center suggests that inappropriate positioning of the loop region caused by destabilization of the loop alters the initial triggering of the nucleophilic attack by Thr267 for the conversion of the precursor protein to the active enzyme. However, it should be noted that the inability of the autocleavage (type 3 phenotype) is caused by various mutations (I75V, L106R, P157L, N235D, and Q299E) in the NylC_p2_-G^122^Y^130^A^36^Q^263^ mutant, as described above (Table 3 and Fig. 1c).

### Proposed model of subunit assembly

The complexity of the oligomeric states of NylC enzymes demonstrates that various association/dissociation steps are involved in the subunit assembly (Fig. 4). We estimated that the major subunit assembly generating the tetramer molecules proceeds *via* A/B dimers (by reactions 1 and 2) for the following reasons: (i) X-ray-crystallographic analyses of wild-type and mutant NylC enzymes revealed that the contact area of the A/B interface was approximately twice that of the A/D interface, suggesting that A/B dimer formation was more predominant than A/D dimer formation; (ii) the A/B dimer was actually identified in an asymmetric unit of the NylC crystals; (iii) the A/D dimer exhibited an irregular shape in which the terminal loop region responsible for contact with the adjacent monomer molecules was exposed to the solvent environment. This looped-out region should be amenable to intracellular proteolytic degradation. In this model, the C/D dimer is equivalent to the A/B dimer in the symmetrical relationship.

Wild-type NylC_*A*_ was largely present as a tetramer, as indicated by the crystallographic and ultracentrifugation analysis (single peak of 4.2 *S* in the sedimentation velocity). In contrast, NylC_p2_ existed at the monomer/dimer/trimer equilibrium in aqueous solution (2.1 *S*, 2.7 *S*, and 3.5 *S*). Thus, it was likely that the partial asymmetry at the A/D interface disrupted tetramer formation (reaction 2) but generated an irregular trimeric structure in aqueous solution by reaction 3 or by successive reactions (reaction 2 followed by reaction 8), although the enzyme was identified to be a tetramer by the crystallographic analysis described above.

Sedimentation velocity analysis of the NylC_p2_-G^122^Y^130^A^36^Q^263^ enzyme showed the major peak (4.5 *S*: tetramer) and the minor peak (2.4 *S*: monomer). The absence of a dimer molecule suggested that the equilibrium of reactions 1 and 2 were optimized to yield tetramers as the major product, leaving monomers as a minor population. Alternatively, the tetrameric structure was coordinately generated from monomers in a single step (reaction 4).

For wild-type NylC_*K*_, the minor peak corresponding to higher oligomers (7.5 *S*) was identified in addition to the major peak of tetramers (4.2 *S*) and the minor peak of monomers (2.1 *S*). This result may imply that further association of the molecules was induced by at least one of the eleven mutations that distinguished NylC_*K*_ and NylC_p2_-G^122^Y^130^A^36^Q^263^ enzymes (Fig. S1).

In conclusion, we revealed that the observed protein stability and oligomeric states are determined by how the monomer unit constituting the oligomeric structure is stabilized due to the subunit interactions in the tetrameric structure. In addition, we demonstrated that the extent of relative stabilization effects at a wider A/B interface on narrower A/D interfaces drastically affects the correct subunit assembly and thermostability and that the amino acid substitutions at specific positions alter not only the protein stability but also the catalytic activity, oligomerization, and processability from the precursor to the active enzyme.

## Methods

### DNA preparation and mutagenesis

The plasmids pSKFC4 (NylC_p2_), pSKRC4 (NylC_*A*_), and pSKKC4 (NylC_*K*_) contained 1.1 kb genes flanked by *Bam*HI and *Pst*I restriction sites, which were cloned into the expression vector pBluescript II SK(+) (Stratagene, La Jolla, CA.)^9^. *E*. *coli* JM109 competent cells were prepared by the conventional CaCl_2_ method^9^ and stored at −80 °C for later use.

Site-directed mutagenesis was performed using a PrimeSTAR Mutagenesis Basal Kit (Takara Bio, Inc., Shiga, Japan) with the primers listed in Supplemental Table S4. The plasmids that contained the 1.1 kb fragments with mutated NylC_p2_ were isolated from transformed *E*. *coli* JM109 cells. DNA sequencing confirmed that the desired mutations were introduced into the wild-type *nylC*_*p2*_ sequence.

To construct a random mutant library from the *nylC* gene, pSKFC4–1 (having G^122^Y^130^A^36^Q^263^ mutations in pSKFC4) was initially digested with *Pst*I and *Bam*HI, and the linearized DNA was amplified by PCR in the presence of nucleotide analogues (2, 5, 10, 40, or 70 μM concentrations of both 8-oxo-2′-deoxyguanosine-5′-triphosphate (8-oxo-dGTP) and 6H,8H-3,4-dihydropyrimido(4,5-C)(1,2)oxazin-7-one-8-β-D-2′ -deoxy-ribofuranoside-5′-triphosphate (dPTP)] (Jena Bioscience GmbH, Jena, Germany) using two primers, FE-BamHI and RE-PstI (Table S4)^29,30^. The amplified 1.1 kb fragment containing the *nylC* gene was digested with *Bam*HI and *Pst*I and the 1.1 kb *Bam*HI-*Pst*I fragment was recovered. The plasmid pSKFC4 was also digested with *Bam*HI and *Pst*I, and a 2.6 kb fragment was recovered. To obtain the mutant library, the two fragments were combined by ligation followed by transformation into *E*. *coli* KP3998 using ampicillin resistance as the selection marker^9^.

### Culture, enzyme assay and enzyme purification

The culture of *E*. *coli* clones and subsequent enzyme purification were performed as previously reported^9,11^. In the NylC activity assays, the enzyme solution (0.1 ml) was mixed with an Ahx-cyclic oligomer solution (0.9 ml) [4 mg ml^−1^ of Ahx-cyclic oligomer in 20 mM phosphate buffer (pH 7.3) containing 10% glycerol (buffer A)] and incubated at 30 °C (standard assay conditions). An increase in the concentration of the amino group was found using trinitrobenzene sulfonic acid (TNBS)^9,11^. Kinetic studies were performed under standard assay conditions, with the exception of the different Ahx-cyclic oligomer concentrations used. NylC encoded on the *Arthrobacter* plasmid pOAD2 (NylC_p2_) and its mutant enzymes were expressed in *E*. *coli* JM109 and purified by ammonium sulfate fractionation, anion-exchange column chromatography, and gel-filtration chromatography^11^.

### Analytical centrifugation

The sedimentation velocity and sedimentation equilibrium of each NylC enzyme (0.34 mg ml^−1^) in phosphate buffer were examined at 20 °C using analytical ultracentrifugation. Centrifugation was performed at 40,000 rpm and 14,000 rpm for sedimentation velocity and equilibrium experiments, respectively, by monitoring the absorbance at 280 nm at intervals of 5 min. Analytical ultracentrifugation experiments were performed using a Beckman-Coulter Optima XL-A analytical ultracentrifuge (Fullerton, CA) after pre-centrifugation at 3,000 rpm for 5 min. Double-sector 12-mm-thick charcoal-epon centerpieces and matched quartz windows were used for all experiments. The experimental sedimentation coefficients were corrected to *s*20, *w*, the sedimentation coefficient expressed in terms of the standard state of water at 20 °C, with the van Holde-Weischet method using the software UltraScan 8.0 (www.ultrascan.uthscsa.edu). Frictional ratios and partial specific volumes were also obtained using the UltraScan software^31^.

### Crystallographic analysis

To analyze the molecular basis of protein stabilization, comparative structural analysis of mutant enzymes that differ in thermostability is informative. Previously, based on the three-dimensional structure of NylC_*A*_ determined at 2.0 Å resolution, we identified the structural interactions occurring at the subunit interfaces A/D (D122G, H130Y, and D36A) and A/B (E263Q). However, the crystals of NylC_*A*_ obtained contain 15 monomer molecules in a large unit cell (space group *I*222); unit-cell parameters *a* = 155.86, *b* = 214.45, and *c* = 478.80 Å). The presence of a large number of monomers in a single asymmetric unit is unsuitable for further crystallographic analysis at higher resolutions. Recently, we reported a new crystallization condition of the NylC_p2_ protein to obtain a crystal that belonged to the space group *C*222_1_ with two molecules in an asymmetric unit (unit-cell parameters *a* = 70.84, *b* = 144.90, and *c* = 129.05 Å) (Table S1).

The crystals of NylC_p2_ and its mutants (G^122^, R^122^, K^122^, V^122^, Y^130^, G^122^Y^130^, and G^122^Y^130^A^36^Q^263^) were grown by the sitting-drop vapor-diffusion method with ammonium sulfate as a precipitant in 0.1 M HEPES buffer (pH 7.5) containing 0.2 M NaCl and 25% glycerol^11^. Placing the crystal in a cold nitrogen stream at 100 K, cryocooling was performed for data collection. Diffraction data sets were collected at SPring-8 (Hyogo, Japan) with a beamline BL41XU equipped with an ADSC Q315 CCD detector system. The following parameters were chosen for data collection: wavelength, 1.0000 Å; crystal-to-detector distance, 200 mm; oscillation range per image, 1.0°. Indexing, integration and scaling of reflections were performed using the *HKL*-2000 program package^32^. Diffraction data were collected from the crystal of NylC_p2_ at resolutions of 1.60 Å. The obtained crystal was spindle-shaped, with a *C*-centered orthorhombic space group *C*222_1_ and unit-cell parameters *a* = 70.84, *b* = 144.90, and *c* = 129.05 Å. Diffraction data were collected from the NylC_p2_ crystal at resolutions of 1.60 Å (Table S1). From NylC mutants, diffraction data were collected at resolutions of 2.00 Å (NylC_p2_-G^122^), 1.20 Å (NylC_p2_-R^122^), 1.10 Å (NylC_p2_-K^122^), 1.05 Å (NylC_p2_-V^122^), 1.90 Å (NylC_p2_-Y^130^), 1.39 Å (NylC_p2_-G^122^Y^130^), and 1.03 Å (NylC_p2_-G^122^Y^130^A^36^Q^263^).

The NylC structure was determined by the molecular replacement method using one protomer (monomer A) of the NylC_*A*_ structure (PDB ID: 3AXG) as a template. Rotation and translation search for molecular replacement was performed by PHASER^33^ within the CCP4 program suite (Collaborative Computational Project, Number 4, 1994). There were two molecules in the asymmetric unit. The crystal packing analysis revealed that each of the NylC_p2_ monomers seems to extensively interact with the adjacent monomer related by the crystallographic two-fold axis.

Rigid-body refinement was performed using the coordinates of the initial model to fit the unit cell of the NylC_*A*_ crystal followed by positional and B-factor refinement with the program REFMAC^34^. With several cycles of manual model rebuilding by Coot^35^, the R-factor and R-free values were determined to be, respectively, 16.1% and 19.2% (for NylC_p2_); 17.3% and 22.6% (for NylC_p2_-G^122^); 15.9% and 20.1% (for NylC_p2_-Y^130^); 17.1% and 20.0% (for NylC_p2_-G^122^Y^130^); 12.2% and 14.3% (for NylC_p2_-K^122^); 12.2% and 14.3% (for NylC_p2_-R^122^); 13.3% and 15.2% (for NylC_p2_-V^122^); and 11.6% and 13.4% (for NylC_p2_-G^122^Y^130^A^36^Q^263^). Ramachandran analysis showed 636 residues (96.1%) in favored regions, 36 residues (3.9%) in allowed regions, and 0 residues (0.0%) in outliers for NylC_p2_. No residue was found in the outlying regions.

The structures of the NylC mutants were determined by the molecular replacement method using one protomer (monomer A) of the NylC_p2_ structure as a template, and subsequently, rigid-body refinements were performed. The results of the crystal structure analysis are summarized in Table S1.

The atomic coordinates and structural factors for NylC_p2_ (PDB ID code: 5XYG); NylC_p2_-G^122^ (PDB ID code: 5XYO); NylC_p2_-R^122^ (PDB ID code: 5XYP); NylC_p2_-K^122^ (PDB ID code: 5XYQ); NylC_p2_-V^122^ (PDB ID code: 5XYS); NylC_p2_-Y^130^ (PDB ID code: 5XYT); NylC_p2_-G^122^Y^130^ (PDB ID code: 5Y0L); NylC_p2_-G^122^Y^130^A^36^Q^263^ (PDB ID code: 5Y0M) have been deposited in the Protein Data Bank (http://www.rcsb.org/).

Figures illustrating the three-dimensional structural models were generated using the programs MolFeat (ver. 4.0, FiatLux Co., Tokyo, Japan) and Chimera^36^.

### CD measurements and thermal stability analyses

CD spectra at far-UV wavelengths (200–250 nm) were measured using a spectropolarimeter (Jasco, model J-720WI, Tokyo, Japan). A cuvette with a path length of 1 mm was used for far UV CD measurements. The results are expressed as the mean residue molar ellipticity, [*θ*], which is defined as [*θ*] = 100 (*θ*_obs_ − *θ*_back_) *l*^−1^ *c*^−1^, where *θ*_obs_ is the observed ellipticity in degrees, *θ*_back_ is the observed ellipticity in degrees in the absence of enzyme (or background), *c* is the molar concentration of the residue, and *l* is the length of the light path (in centimeters). The temperature was kept at 25 or 95 °C with a Jasco PTC-348WI Peltier system. For thermal transition curve experiments, the temperature was increased from 25 to 95 °C with a Jasco PTC-348WI Peltier system at 1 °C min^−1^ by monitoring the CD signal at 220 nm. The protein concentration used was 0.1 mg ml^−1^.

*T*_m_ and Δ*H* were determined by regression analysis using nonlinear least-squares fitting of data to a sigmoidal equation under the assumption of a two-state transition between the folded and heat-denatured states^37^. We performed thermodynamic analyses, although the thermal unfolding of proteins was not confirmed. The following equation describes the signal intensity monitored by CD (the observed ellipticity in degrees).

$${\theta }_{{\rm{obs}}}=\frac{(a-c)+(b-d)T}{1+\exp (-\frac{{\rm{\Delta }}H\,({T}_{{\rm{m}}})}{R}(\frac{1}{T}-\frac{1}{{T}_{{\rm{m}}}})+\frac{{\rm{\Delta }}{C}_{{\rm{p}}}}{R}(\frac{{T}_{m}}{T}-1+\,\mathrm{ln}\,\frac{T}{{T}_{{\rm{m}}}}))}+(c\,+\,dT)$$

where *θ*_obs_ is the signal intensity monitored by CD; the pre- and post-unfolding baselines are described by *a* + *bT* and *c* + *dT*, respectively; *T*, *T*_m_, and *R* indicate the temperature, the midpoint temperature of denaturation, and the gas constant, respectively; the change in enthalpy for the global unfolding of proteins is represented by Δ*H*; and the change in heat capacity is shown by Δ*C*_p_.

### Quantitative reverse-transcription-PCR (qRT-PCR)

Total RNA was extracted from the cells using a PureLink^TM^ RNA Mini Kit (Thermo Fisher Scientific, Inc., Waltham, U.S.A.) according to the manufacturer’s instructions, and qRT-PCR analysis was performed using a One Step SYBR^®^ PrimeScript™ RT-PCR Kit II (Takara Bio, Inc. Shiga, Japan), a Bio-Rad MiniOpticon Real-Time PCR system (Bio-Rad, Inc., Hercules, U.S.A.) and Bio-Rad’s Real-Time PCR system (MiniOpticon, Bio-Rad Japan, Tokyo, Japan). Two primers (FE-BamHI and RE-PstI) were used for cDNA synthesis and amplification of the *nylC* gene (Table S5). A typical reaction mixture for qRT-PCR contained template RNA (50 ng), 2x One Step SYBR RT-PCR Buffer 4 (Takara Bio, Inc.) (12.5 µl), PrimeScript One Step Enzyme Mix 2 (Takara Bio, Inc.) (1.0 µl), each primer (0.4 µM), and RNase-free H_2_O (up to 25 µl). The thermal program employed was as follows: initial denaturation at 42 °C for 5 s, 95 °C for 10 s, 40 cycles of denaturation at 95 °C for 5 s, annealing and extension at 60 °C for 30 s, and the melting-curve step. The gene expression level was evaluated as the relative amounts of a specific RNA calculated from the qRT-PCR data using the 2^−ΔΔCT^ method^38^.

### Immunological detection of NylC antigenic protein

#### Antibody preparation

Polyclonal antibody against purified NylC_p2_-G^122^Y^130^A^36^Q^263^ was ordered from Eurofins Genomics (Tokyo, Japan). Briefly, anti-NylC serum was prepared by subcutaneous inoculation of rabbits with the antigen emulsified in Freund’s complete adjuvant. Rabbits were bled after four injections (0.2–0.3 mg antigen per injection, total 0.9 mg).

#### Western blot analysis

After *E*. *coli* cells were lysed by sonication and centrifuged (10,000 × g for 5 min), the cell extracts (soluble fraction) and precipitates (insoluble fraction) were obtained. These samples were boiled in SDS solution and fractionated by SDS-PAGE (17.5%). Protein bands were detected by Coomassie brilliant blue staining. For immunological detection, the proteins contained in the gels were electrophoretically transferred to Immobilon PVDF Transfer Membranes (Pore Size: 0.45 µm, Millipore) using a blotting instrument (Atto, model AE-7500). NylC-antigenic proteins were detected by immunoassay using polyclonal antibody against purified NylC_p2_-G^122^Y^130^A^36^Q^263^ and goat anti-rabbit IgG H&L (HRP) (Abcam, Cambridge, U.K.)^39^.

#### Sandwich ELISA

Standard ELISA was conducted by the conventional method with modifications. Briefly, 100 µl of the anti-NylC serum (10 µg ml^−1^) was dispensed into each well of a 96-well microtiter plate (MaxiSorp, Nunc), and the plate was left overnight in a refrigerator for immobilization. After removing the solution and washing, blocking treatment was performed, and the amount of NylC antigenic protein was quantified using a Protein Detector ELISA Kit (SeraCare Life Science, Massachusetts, U.S.A.). The samples were automatically analyzed by measuring the absorbance at 405 nm using a Crocodile Mini-Workstation (Titertek-Berthold, Pforzheim, Germany). Standard NylC solutions were prepared using purified NylC_p2_-G^122^Y^130^A^36^Q^263^ enzyme (up to 10 µg l^−1^) by diluting the stock solution with the coating solution. The amount of the NylC-antigenic protein was estimated from the calibration curve.

## Electronic supplementary material

On-line Supplementary Information
