# Supplementary material for: Structural basis of the correct subunit assembly, aggregation, and intracellular degradation of nylon hydrolase
Source: Sci Rep. 2018 Jun 27;8:9725. doi: 10.1038/s41598-018-27860-w (PMC6021441; doi:10.1038/s41598-018-27860-w)
Supplement: Supplementary file 1 — On-line Supplementary Information [file 41598_2018_27860_MOESM1_ESM.pdf]

Supplementary information

Structural basis of the correct subunit assembly, aggregation, and intracellular degradation of nylon hydrolase

Running title: Protein stability and subunit assembly of nylon hydrolase

Seiji Negoro[1*], Naoki Shibata[2,3*], Young-Ho Lee[4*], Ikki Takehara[1], Ryo Kinugasa[1], Keisuke Nagai[1], Yusuke Tanaka[1], Dai-ichiro Kato[5], Masahiro Takeo[1], Yuji Goto[4], and Yoshiki Higuchi[2,3]

[1]Department of Applied Chemistry, Graduate School of Engineering, University of Hyogo, Hyogo 671-2280; [2]Department of Picobiology, Graduate School of Life Science, University of Hyogo, Hyogo 678-1297; [3]RIKEN Harima Institute, SPring-8 Center, Hyogo 679-5148; [4]Institute for Protein Research, Osaka University, Osaka 565-0871, Japan, [5]Graduate School of Science and Engineering, Kagoshima University

SUPPLEMENTARY TABLES

Table S1 (A). Data Collection statistics

Data collection	$NylC_{p2}$	G^{122}	Y^{130}	$G^{122}Y^{130}$
X-ray source	BL41XU, SPring-8	Rigaku, MicroMax 007	Rigaku, MicroMax 007	NW12A, PhotonFactory
Detector	ADSC QUANTUM 315	RIGAKU RAXIS VII	RIGAKU RAXIS VII	ADSC QUANTUM 210
Space group	$C222_1$	$C222_1$	$C222_1$	$C222_1$
Unit cell (Å) *a*	70.84	70.48	70.23	69.88
b	144.90	144.46	144.62	144.00
c	129.05	128.35	128.18	129.28
Wavelength (Å)	1.0000	1.0000	1.0000	1.0000
Resolution (outer shell) (Å)	50 – 1.60 (1.64 – 1.60)	50 – 2.00 (2.07 – 2.00)	50 – 1.90 (1.97 – 1.90)	50 – 1.39 (1.41 – 1.39)
Total reflections	598,801	121533	138951	900,529
Unique reflections (outer shell)	86,374 (5,510)	43634	49919	128,858 (6,285)
Completeness (outer shell) (%)	99.5 (96.3)	97.8 (84.0)	96.5 (75.6)	99.1 (98.0)
*R_{merge} (outer shell) (%)	5.5 (45.1)	5.2 (19.0)	4.0 (12.4)	4.7 (88.6)
$\langle I / \sigma(I) \rangle$ (outer shell)	18.3 (4.1)	12.9 (4.2)	14.9 (6.0)	38.5 (2.0)
$CC_{1/2}$	0.999 (0.909)	0.999 (0.943)	0.999 (0.982)	0.997 (0.826)
Multiplicity	6.9 (5.9)	2.79 (2.10)	2.78 (2.05)	7.0 (6.3)
Refinement				
Resolution (outer shell) (Å)	50 – 1.60 (1.65 –1.60)	50 – 2.00 (2.05 – 2.00)	50 – 1.90 (1.95 – 1.90)	50 – 1.39 (1.42 –1.39)
R_{work} (outer shell) (%)	16.1 (23.1)	17.3 (26.4)	15.9 (21.9)	17.1 (29.6)
R_{free} (outer shell) (%)	19.2 (25.3)	22.6 (31.1)	20.1 (25.9)	20.0 (31.6)
R.m.s. deviations from ideal values, Bond lengths (Å)/bond angles (°)	0.015/1.5	0.006/1.2	0.009/1.0	0.015/1.7
No. of atoms				
Protein	4847	4858	4866	4890
Solvent	421	317	390	632
Others	32	18	17	19
Ramachandran favored (%)	96.1	95.4	96.4	95.8
Ramachandran allowed (%)	3.9	4.6	3.3	4.2
Ramachandran outliers (%)	0.0	0.0	0.3	0.0

$R = \sum_{hkl} \| |F_{obs}| - k |F_{calc}| \| (\sum_{hkl} |F_{obs}|)^{-1}$, k : scaling factor
Values in parentheses are for the outer resolution shell

Table S1 (B). Data Collection statistics

Data collection		$G^{122}Y^{130}$-$A^{36}Q^{263}$	V^{122}	K^{122}	R^{122}
X-ray source		BL44XU, SPring-8	BL-1A, PhotonFactory	BL-1A, PhotonFactory	BL-1A, PhotonFactory
Detector		RAYONIX MX300HE	DECTRIS PILATUS 2M	DECTRIS PILATUS 2M	DECTRIS PILATUS 2M
Space group		$C222_1$	$C222_1$	$C222_1$	$C222_1$
Unit cell (Å)	*a*	70.42	70.46	70.66	70.25
	b	144.42	144.78	144.78	145.11
	c	128.27	128.57	128.19	128.56
Wavelength (Å)		0.9000	1.0000	1.0000	1.0000
Resolution (outer shell) (Å)		50 – 1.03 (1.04 – 1.03)	50 – 1.05 (1.07 – 1.05)	50 – 1.10 (1.12 – 1.10)	50 – 1.20 (1.22 – 1.20)
Total reflections		3,098,846	2,549,661	1,638,935	1,663,275
Unique reflections (outer shell)		318,913 (12,557)	300,775 (12,735)	264,009 (13,089)	203,670 (10,037)
Completeness (outer shell) (%)		99.9 (99.3)	98.7 (84.3)	99.9 (99.5)	99.8 (98.9)
*R_{merge} (outer shell) (%)		7.0 (94.1)	6.2 (61.2)	7.9(72.0)	8.1 (44.4)
$\langle I / \sigma(I) \rangle$ (outer shell)		41.9 (2.39)	34.7 (1.85)	21.2 (2.12)	29.5 (2.97)
CC1/2		0.999 (0.712)	0.999 (0.710)	0.994 (0.722)	0.995 (0.853)
Multiplicity		9.7 (6.8)	8.5 (3.6)	6.2 (5.0)	8.2 (4.2)
Refinement					
Resolution (outer shell) (Å)		50 – 1.03 (1.06 – 1.03)	50 – 1.05 (1.08 – 1.05)	50 – 1.10 (1.13 – 1.10)	50 – 1.20 (1.23 – 1.20)
R_{work} (outer shell) (%)		11.6 (21.6)	13.3 (25.4)	12.2 (18.7)	12.2 (13.4)
R_{free} (outer shell) (%)		13.4 (23.0)	15.2 (27.5)	14.3 (20.3)	14.3 (17.1)
R.m.s. deviations from ideal values, Bond lengths (Å)/bond angles (°)		0.019/2.0	0.019/2.0	0.012/1.4	0.013/1.5
No. of atoms					
Protein		4844	4871	4875	4879
Solvent		595	611	669	595
Others		86	22	34	21
Ramachandran most favored (%)		95.4	96.1	96.0	96.3
Ramachandran allowed (%)		4.5	3.9	4.0	3.7
Ramachandran outliers (%)		0.1	0.0	0.0	0.0

$R = \sum_{hkl} | |F_{obs}| - k |F_{calc}| | (\sum_{hkl} |F_{obs}|)^{-1}$, k : scaling factor
Values in parentheses are for the outer resolution shell

Table S2. Distances between α-carbon of the two selected amino acids at two helix α1 located at the subunit interface

Measured residues((A) – (D))	Distance (Å)				
	NylC$_{p2}$ (T_m = 52.9 °C)	Arg122 (T_m = 69.5 °C)	Lys122 (T_m = 70.8 °C)	Val122 (T_m = 74.7 °C)	Gly122 (T_m = 75.1 °C)
112 - 122	7.04	6.87	6.98	6.75	6.77
115 - 118	5.67	5.57	5.62	5.58	5.44
118 - 115	5.76	5.51	5.53	5.61	5.45
122 – 112	8.43	6.89	6.87	6.64	6.63

Table S3. Nearest distances between the side chain atoms of the two selected amino acids located at the subunit interface

Measured residues	Distance (Å)				
	NylC$_{p2}$ (T_m = 52.9 °C)	Arg122 (T_m = 69.5 °C)	Lys122 (T_m = 70.8 °C)	Val122 (T_m = 74.7 °C)	Gly122 (T_m = 75.1 °C)
K159-NZ(A) E115-OE1(D)	2.87<br>3.14	2.76<br>3.48	2.63 - 2.75<br>3.16 - 4.0	2.71<br>3.46	2.65<br>3.39
K159-NZ(D) E115-OE1(A)	2.79<br>3.60	2.69<br>3.43	2.71<br>3.36	2.70<br>3.44	2.83<br>3.43

Table S4 (A). PCR primers for site-directed mutagenesis of *nylC* gene

Oligonucleotide	5' Sequence of oligonucleotide 3'
F D122G	GGGGTGAGCGGCGCGCTCCTGGAACGCCTC
R D122G	TTCCAGGAGCGCGCCGCTCACCCCGGCGCC
F D122NNN	GGGGTGAGCNNNGCGCTCCTGGAACGCCTC
R D122NNN	TTCCAGGAGCGCNNNGCTCACCCCGGCGCC
F L137A	GCCGAGGCCCAGCTGGTGTCGTCGGCG
R L137A	CAGCTGGGCCTCGGCGAAGCCGGTGCG
F Q299NNN	GGCATCNNNCCGTTCCACACCGACATGGACGGCGAC
R Q299NNN	GGACGGNNNGATGCCGCGGTGCATCGAACTGTG

Table S4 (B). PCR primers for amplification of *nylC* gene

Oligonucleotide	5' Sequence of oligonucleotide 3'
FE-BamH	AACTAGTGGATCCCTACTCGCA
RE-PstI	ACTGAATTCCTGCAGAGCGTTCC

SUPPLEMENTARY FIGURES

Fig. S1. Comparison of the amino acid sequences of NylC from *Arthrobacter* plasmid pOAD2 ($NylC_{p2}$), *Agromyces* ($NylC_A$), *Kocuria* ($NylC_K$) and $NylC_{p2}$-GYAQ. The amino acid residues are shown as *one-letter* codes. Fifteen residues differed between $NylC_{p2}$ and $NylC_K$, and the five residues in *green* boxes are altered in both $NylC_K$ and $NylC_A$. The ten residues in *blue* boxes are unique to $NylC_K$. The $NylC_{p2}$-GYAQ mutant contains four amino acid substitutions, in which $NylC_A$-type (D122G and H130Y) and $NylC_K$-type (D36A and E263Q) substitutions are integrated in the sequence of $NylC_{p2}$.

Fig. S2. CD spectra of various NylC mutants. ***a and b.*** CD spectra were measured at 25 °C (***a***) and 95 °C (***b***). ***c.*** Thermal transition curves of the various NylC mutant enzymes. CD measurements were performed at 220 nm from 25 to 95 °C (1 °C min^{-1}). The results are expressed as the mean residue molar ellipticity [θ]. ***d***. T_m (melting temperature of heat denaturation) and ΔH (change in enthalpy for the global unfolding of proteins) were determined.

Fig. S3. Thermostability of various NylC mutants. Enzyme solutions (1 mg ml^{-1}) were incubated at various temperatures for 30 min, and the residual activity was assayed at 30 °C.

Fig. S4. Western blot analysis of various NylC mutants. Soluble fraction: After the cell extracts of *E. coli* clones obtained by sonication were fractionated by SDS-PAGE (17.5%), whole protein bands (upper panel) and NylC-antigenic proteins (lower panel) were analyzed. Insoluble fraction: After the precipitates obtained by centrifugation of the sonicated cells were boiled in SDS solution and fractionated by SDS-PAGE, whole proteins and NylC-antigenic proteins were similarly analyzed (see "Materials and Methods"). ***a.*** Cell extracts of *E. coli* expressing $NylC_{p2}$-$G^{122}Y^{130}A^{36}Q^{263}$ (lane 1), $NylC_{p2}$-P^{122} (lane 2), $NylC_{p2}$-H^{122} (lane 3), $NylC_{p2}$-W^{122} (lane 4), $NylC_{p2}$-$G^{122}Y^{130}A^{36}Q^{263}$-$T^{179}$ (lane 5), $NylC_{p2}$-$G^{122}Y^{130}A^{36}Q^{263}$-$T^{295}$ (lane 6), $NylC_{p2}$-$G^{122}Y^{130}A^{36}Q^{263}$-$D^{299}$ (lane 7), and $NylC_{p2}$-$G^{122}Y^{130}A^{36}Q^{263}$-$E^{299}$ (lane 8). ***b.*** Cell extracts of *E. coli* expressing $NylC_{p2}$-$G^{122}Y^{130}A^{36}Q^{263}$ (lane 1), $NylC_{p2}$-$G^{122}Y^{130}A^{36}Q^{263}$-$V^{75}$ (lane 2), $NylC_{p2}$-$G^{122}Y^{130}A^{36}Q^{263}$-$R^{106}$ (lane 3), $NylC_{p2}$-$G^{122}Y^{130}A^{36}Q^{263}$-$L^{141}$ (lane 4), $NylC_{p2}$-$G^{122}Y^{130}A^{36}Q^{263}$-$L^{157}$ (lane 5), $NylC_{p2}$-$G^{122}Y^{130}A^{36}Q^{263}$-$N^{191}$ (lane 6), and $NylC_{p2}$-$G^{122}Y^{130}A^{36}Q^{263}$-$D^{235}$ (lane 7). Lane M, molecular size markers.

Fig. S5. Elution profiles from ion exchange chromatography. Cell extracts of *E. coli* clones expressing the various *nylC* mutants were applied to a DEAE-Sepharose column. The elution profiles of type 1 mutants (Glu122 mutant) and type 4 mutants (His122, Trp122, and Pro122) are compared.

Fig. S6. Comparison of the backbone structures of $NylC_{p2}$, $NylC_A$ and $NylC_{p2}$-$G^{122}Y^{130}A^{36}Q^{263}$. ***a.*** The structures of $NylC_{p2}$ (*orange*), $NylC_A$ (*gray*), and $NylC_{p2}$-$G^{122}Y^{130}A^{36}Q^{263}$ (*blue*) superimposed using Chimera (1) are shown as stereoviews. ***b***. Root mean square deviations for main-chain atoms calculated by the program CNS (2) are plotted for the amino acid residue numbers. *Orange*, deviations between $NylC_A$ and $NylC_{p2}$. *Blue*, deviations between $NylC_A$ and $NylC_{p2}$-$G^{122}Y^{130}A^{36}Q^{263}$. Positions of loop (L1-L7) and 3_{10}-helix (η3) regions are shown.

Fig. S7. Temperature factors for $NylC_{p2}$ and $NylC_{p2}$-$G^{122}Y^{130}$. The average values for C_α, C, and N in each amino acid residue in monomer A (upper) and monomer D (lower) are plotted as a function of residue number. *Orange*, wild-type $NylC_{p2}$; *green*, $NylC_{p2}$-$G^{122}Y^{130}$. The positions of seven loop regions (L1-L7) are shown as *red* bars.

Fig. S8. Polyacryl amide gel electrophoresis of wild-type and mutant enzymes. *a*. SDS-PAGE (17.5% gel) of purified NylC. Lane M, molecular size marker; Lane 1, $NylC_{A}$; Lane 2, $NylC_{K}$; Lane 3, $NylC_{p2}$; Lane 4, $NylC_{p2}$-K^{122}; Lane 5, $NylC_{p2}$-L^{122}; Lane 6, $NylC_{p2}$-R^{122}; Lane 7, $NylC_{p2}$-V^{122}; Lane 8, $NylC_{p2}$-Q^{122}; Lane 9, $NylC_{p2}$-N^{122}; Lane 10, $NylC_{p2}$-$G^{122}Y^{130}A^{36}Q^{263}$; Lane 11, $NylC_{p2}$-A^{137}; Lane 12, $NylC_{p2}$-$G^{122}Y^{130}$; Lane 13, $NylC_{p2}$-$G^{122}A^{137}$; Lane 14, $NylC_{p2}$-G^{122}; Lane 15, $NylC_{p2}$-Y^{130}. ***b*.** Native-PAGE (10% gel) of purified NylC. Lane 1, $NylC_{A}$; Lane 2, $NylC_{K}$; Lane 3, $NylC_{p2}$; Lane 4, $NylC_{p2}$-L^{122}; Lane 5, $NylC_{p2}$-N^{122}; Lane 6, $NylC_{p2}$-Q^{122}; Lane 7, $NylC_{p2}$-R^{122}; Lane 8, $NylC_{p2}$-K^{122}; Lane 9, $NylC_{p2}$-V^{122}; Lane 10, $NylC_{p2}$-G^{122}; Lane 11, $NylC_{p2}$-$G^{122}Y^{130}A^{36}Q^{263}$; Lane 12, $NylC_{p2}$-$G^{122}Y^{130}$; Lane 13, $NylC_{p2}$-A^{137}; and Lane 14, $NylC_{p2}$-$G^{122}A^{137}$.

REFERENCES

1. Pettersen EF, Goddard TD, Huang CC, Couch GS, Greenblatt DM, Meng EC & Ferrin TE. UCSF Chimera - A Visualization System for Exploratory Research and Analysis. *J. Comput. Chem.* **25**, 1605-1612 (2004).
2. Brunger AT, Adams PD, Clore GM, DeLano WL, Gros P, Grosse-Kunstleve RW, Jiang JS, Kuszewski J, Nilges N, Pannu NS, Read RJ, Rice LM, Simonson T & Warren GL Crystallography and NMR system (CNS): a new software system for macromolecular structure determination. *Acta Cryst.* **D54**, 905-921 (1998).

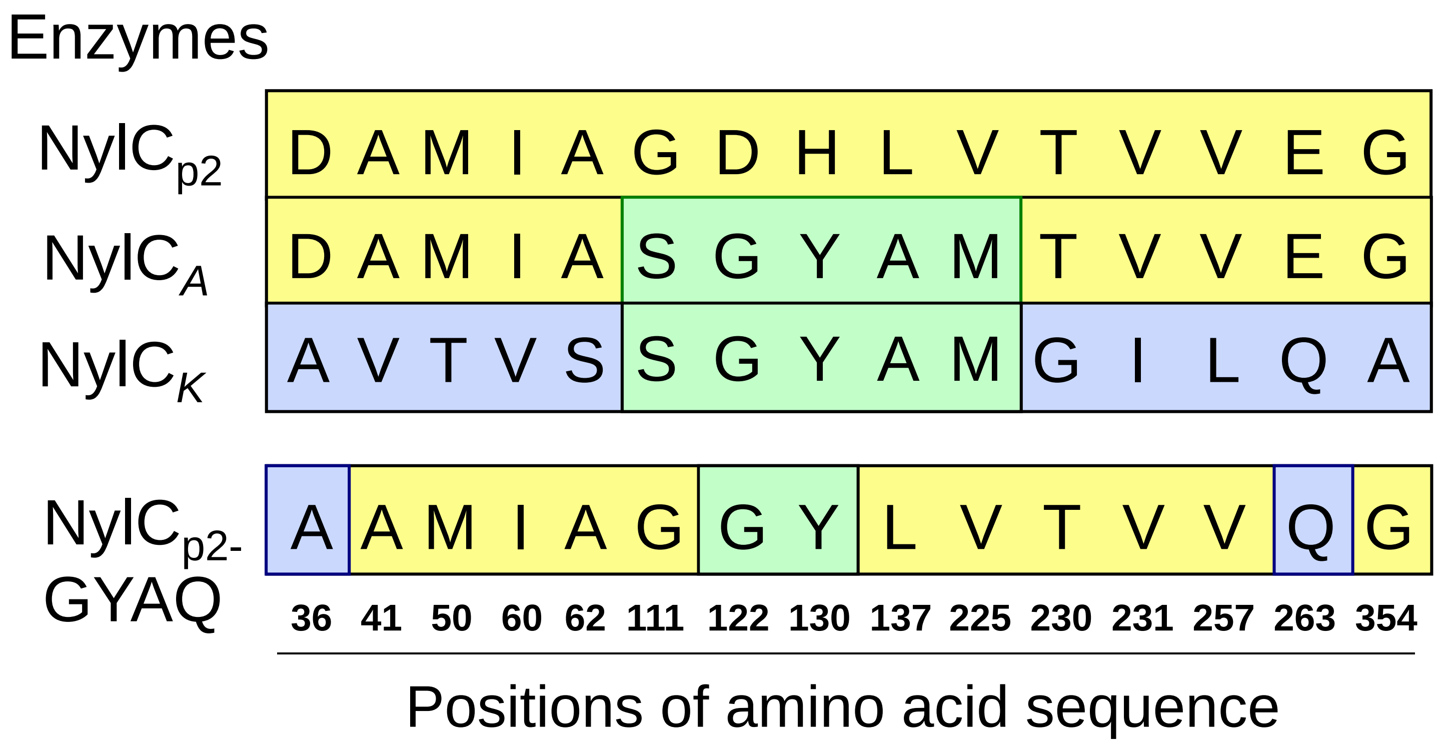
Enzymes
NylCp2
D A M I A G D H L V T V V E G
NylCA
D A M I A S G Y A M T V V E G
NylCK
A V T V S S G Y A M G I L Q A
NylCp2-GYAQ
A A M I A G G Y L V T V V Q G
36 41 50 60 62 111 122 130 137 225 230 231 257 263 354
Positions of amino acid sequence

Fig. S1

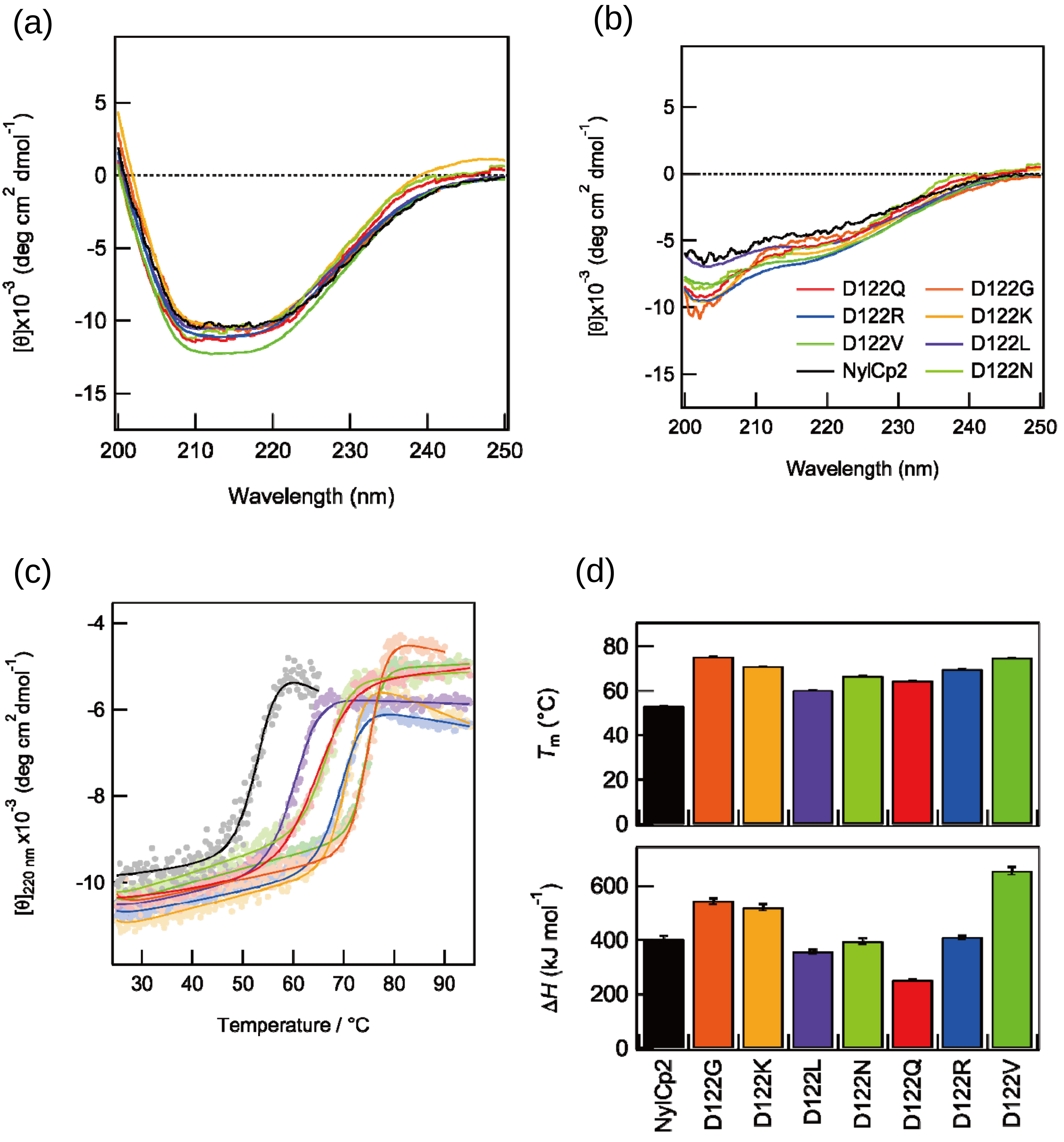

(a)
[θ]x10^-3 (deg cm^2 dmol^-1)
5
0
-5
-10
-15
200
210
220
230
240
250
Wavelength (nm)
(b)
[θ]x10^-3 (deg cm^2 dmol^-1)
D122Q
D122G
D122R
D122K
D122V
D122L
NylCp2
D122N
Wavelength (nm)
(c)
[θ]220 nm x10^-3 (deg cm^2 dmol^-1)
-4
-6
-8
-10
30
40
50
60
70
80
90
Temperature / °C
(d)
Tm (°C)
ΔH (kJ mol^-1)
NylCp2
D122G
D122K
D122L
D122N
D122Q
D122R
D122V

Fig. S2

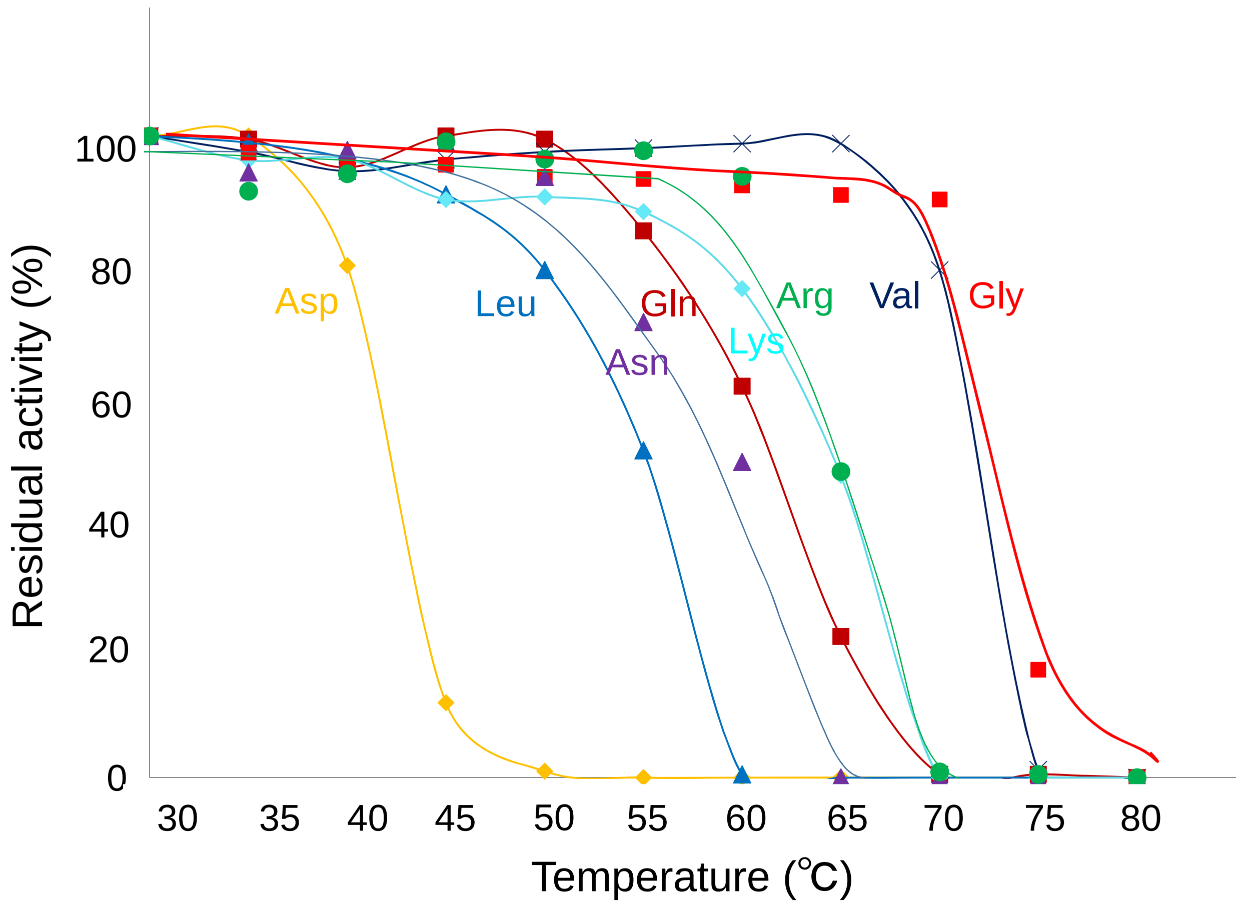
Residual activity (%)
100
80
60
40
20
0
30
35
40
45
50
55
60
65
70
75
80
Temperature (℃)
Asp
Leu
Gln
Asn
Lys
Arg
Val
Gly

Fig. S3

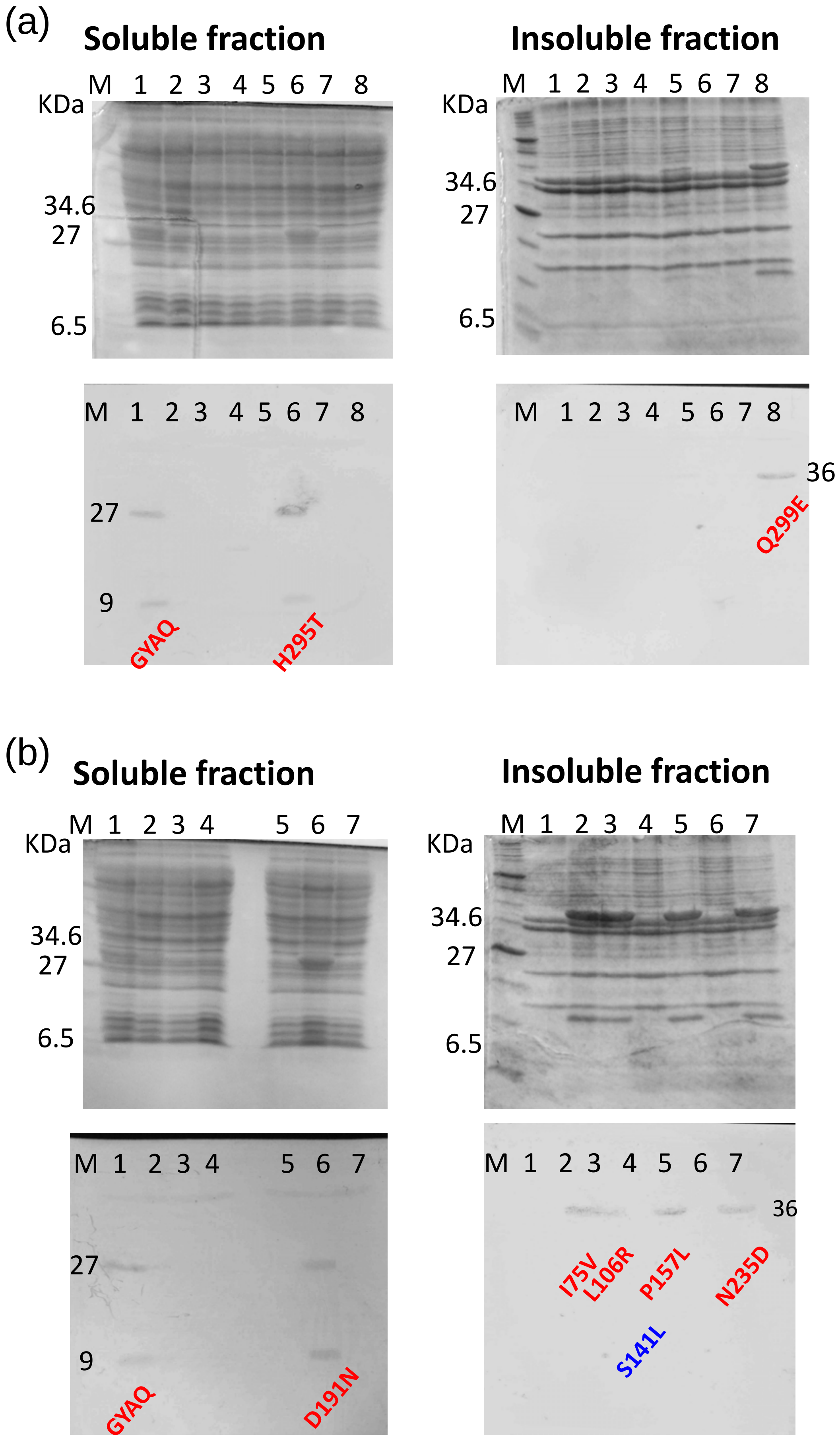
(a)
Soluble fraction
Insoluble fraction
M 1 2 3 4 5 6 7 8
KDa
34.6
27
6.5
M 1 2 3 4 5 6 7 8
27
9
GYAQ
H295T
36
Q299E
(b)
Soluble fraction
Insoluble fraction
M 1 2 3 4 5 6 7
KDa
34.6
27
6.5
27
9
GYAQ
D191N
36
I75V
L106R
P157L
N235D
S141L

Fig. S4

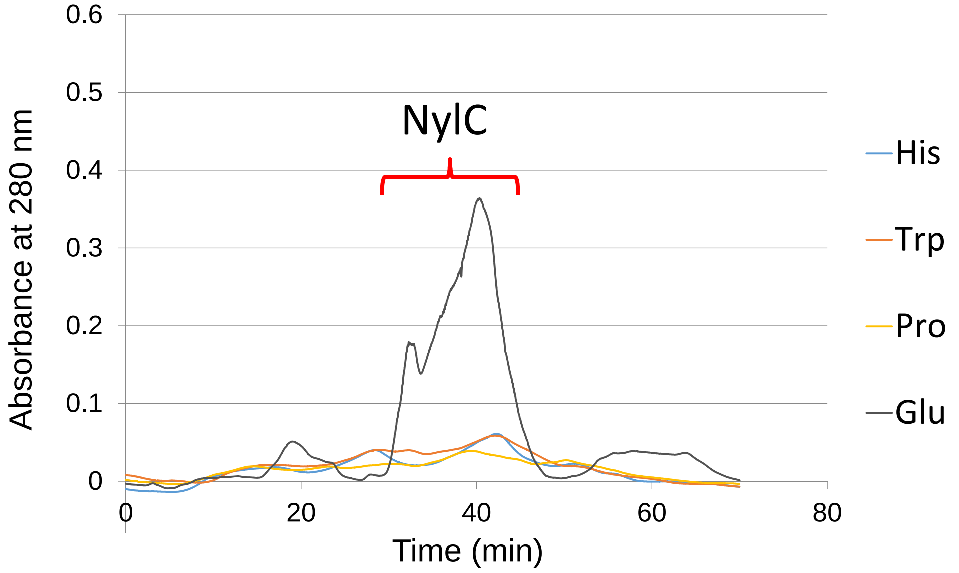
NylC
0.6
0.5
0.4
0.3
0.2
0.1
0
Absorbance at 280 nm
0
20
40
60
80
Time (min)
His
Trp
Pro
Glu

Fig. S5

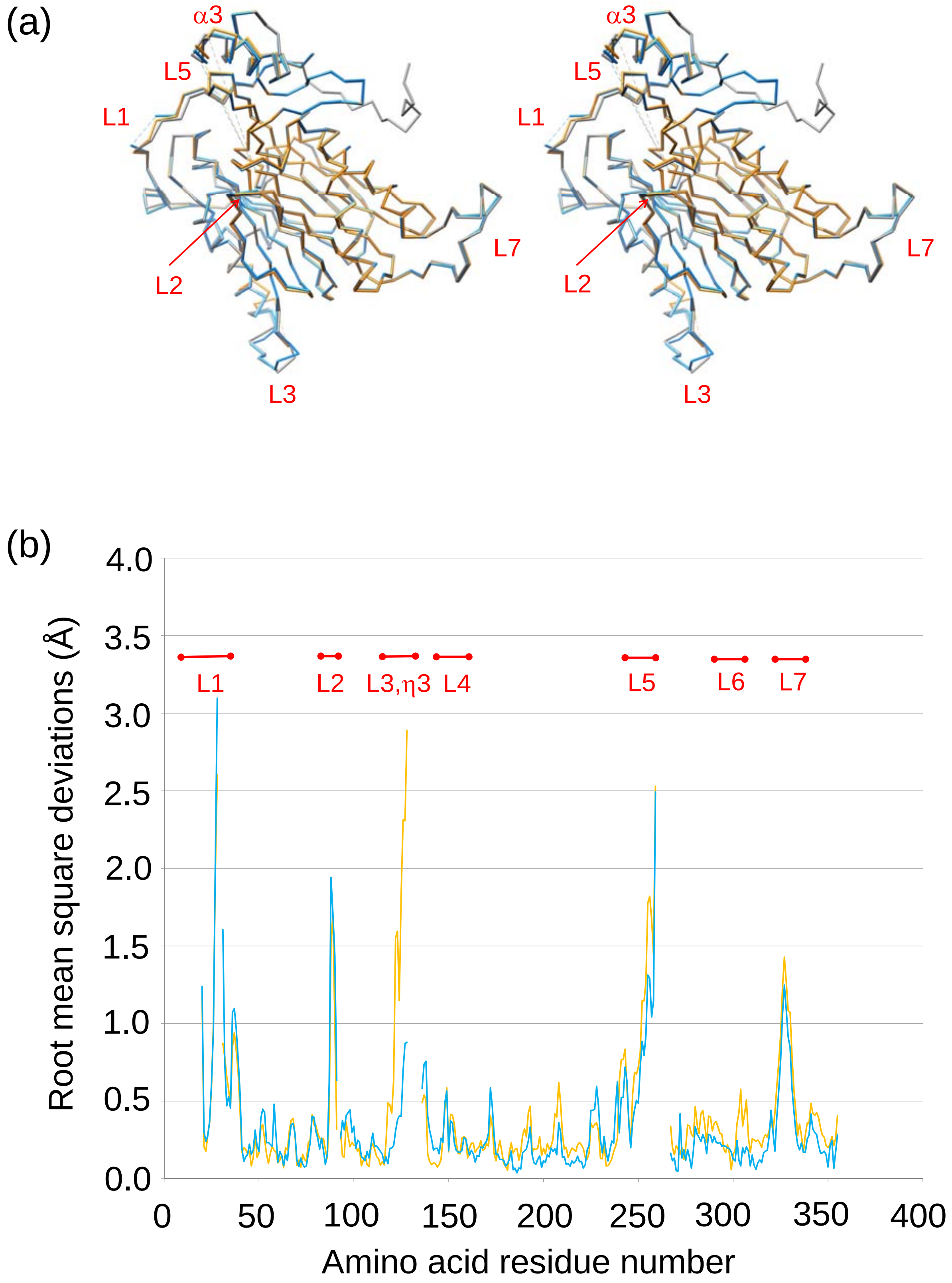
(a)
α3
L5
L1
L2
L7
L3
α3
L5
L1
L2
L7
L3
(b)
4.0
3.5
3.0
2.5
2.0
1.5
1.0
0.5
0.0
Root mean square deviations (Å)
L1
L2
L3,η3
L4
L5
L6
L7
0
50
100
150
200
250
300
350
400
Amino acid residue number

Fig. S6

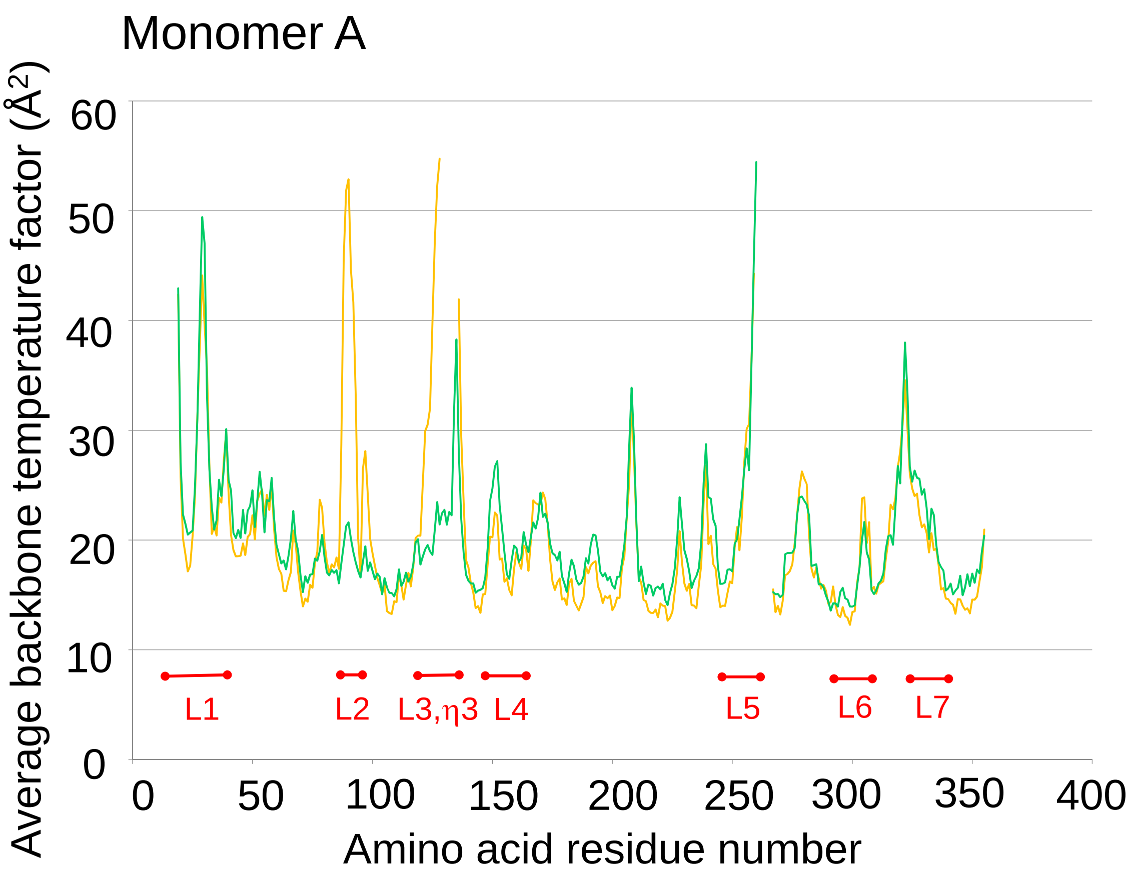
Monomer A
Average backbone temperature factor (Å²)
60
50
40
30
20
10
0
L1
L2
L3,η3
L4
L5
L6
L7
0
50
100
150
200
250
300
350
400
Amino acid residue number

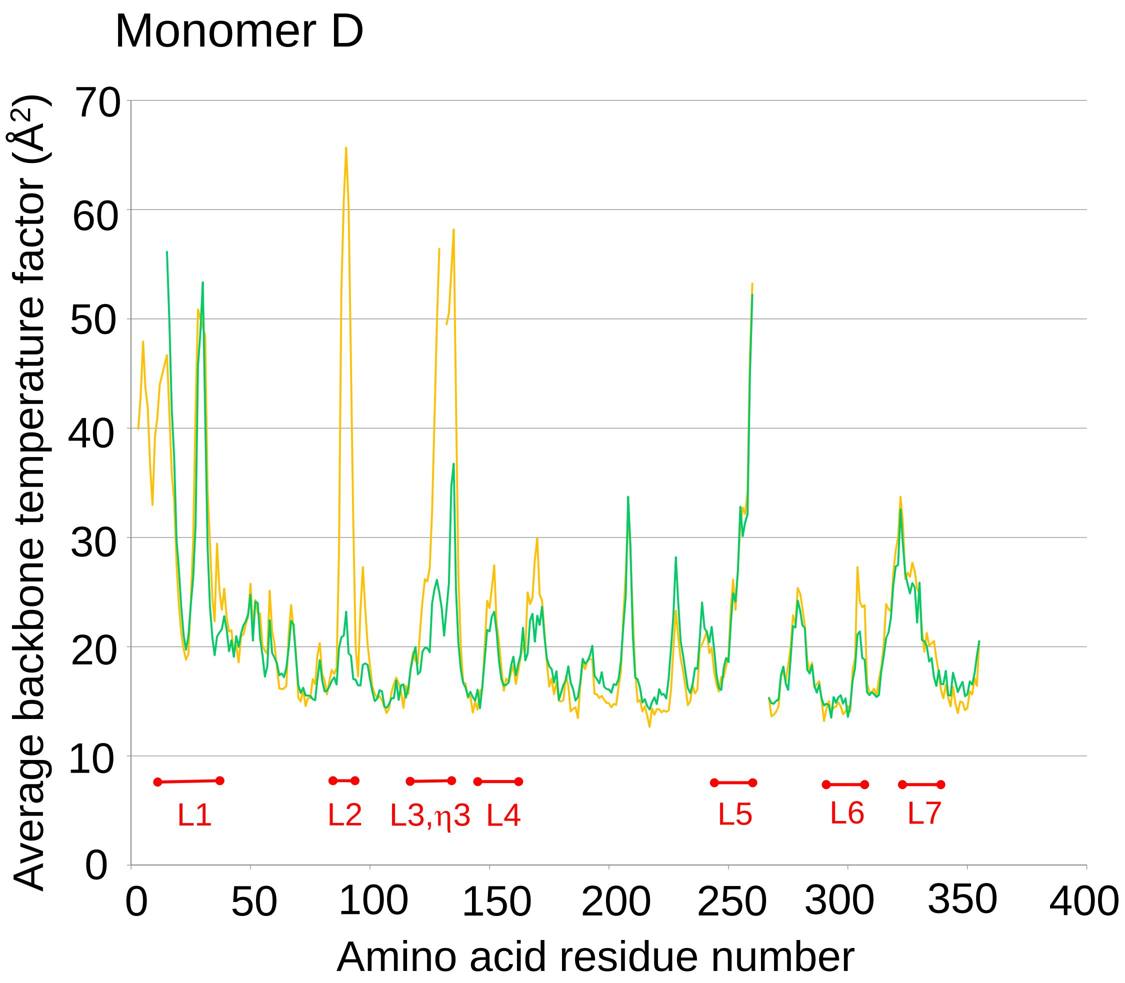
Monomer D
Average backbone temperature factor (Å²)
70
60
50
40
30
20
10
0
L1
L2
L3,η3
L4
L5
L6
L7
0
50
100
150
200
250
300
350
400
Amino acid residue number

Fig. S7

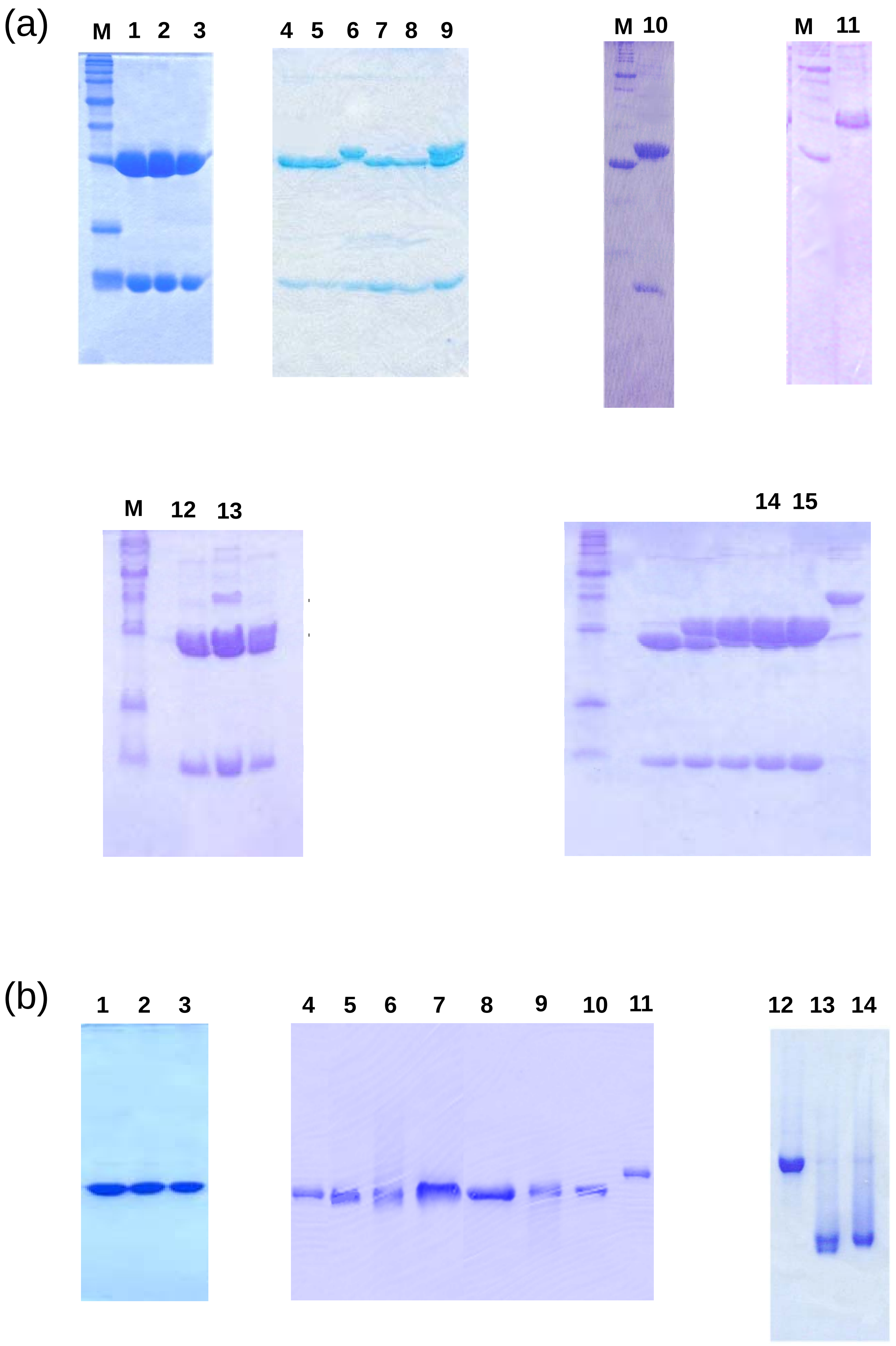

Fig. S8
